# Surrogate infection model predicts optimal alveolar macrophage number for clearance of *Aspergillus fumigatus* infections

**DOI:** 10.1038/s41540-023-00272-x

**Published:** 2023-04-10

**Authors:** Christoph Saffer, Sandra Timme, Paul Rudolph, Marc Thilo Figge

**Affiliations:** 1 Research Group Applied Systems Biology, Leibniz Institute for Natural Product Research and Infection Biology – Hans Knöll Institute, Jena, Germany; 2 Faculty of Biological Sciences, Friedrich Schiller University Jena, Jena, Germany; 3 Institute of Microbiology, Faculty of Biological Sciences, Friedrich Schiller University Jena, Jena, Germany

**Keywords:** Computer modelling, Numerical simulations, Dynamical systems

## Abstract

The immune system has to fight off hundreds of microbial invaders every day, such as the human-pathogenic fungus *Aspergillus fumigatus*. The fungal conidia can reach the lower respiratory tract, swell and form hyphae within six hours causing life-threatening invasive aspergillosis. Invading pathogens are continuously recognized and eliminated by alveolar macrophages (AM). Their number plays an essential role, but remains controversial with measurements varying by a factor greater than ten for the human lung. We here investigate the impact of the AM number on the clearance of *A. fumigatus* conidia in humans and mice using analytical and numerical modeling approaches. A three-dimensional to-scale hybrid agent-based model (hABM) of the human and murine alveolus allowed us to simulate millions of virtual infection scenarios, and to gain quantitative insights into the infection dynamics for varying AM numbers and infection doses. Since hABM simulations are computationally expensive, we derived and trained an analytical surrogate infection model on the large dataset of numerical simulations. This enables reducing the number of hABM simulations while still providing (i) accurate and immediate predictions on infection progression, (ii) quantitative hypotheses on the infection dynamics under healthy and immunocompromised conditions, and (iii) optimal AM numbers for combating *A. fumigatus* infections in humans and mice.

## Introduction

The ubiquitous mold *Aspergillus fumigatus* is the most important airborne human-pathogenic fungus^1^. While immunocompetent individuals are usually not affected by *A. fumigatus*, immunocompromised patients can develop life-threatening infections such as invasive aspergillosis with mortality rates up to 90%^2–5^. Every individual inhales up to thousand conidia of *A. fumigatus* every day^6^, which—due to their small size of approximately 2–3 µm^7,8^—can pass through the upper respiratory tract and reach the alveoli in the lower airways^9–11^.

There, the conidia are embedded in the moist and nutrient-rich surfactant layer covering the alveolar epithelial cells (AEC), allowing them to swell and germinate within a short period of about six hours^7,12^. Furthermore, they come into immediate contact with epithelial cells that are known to be able to internalize conidia^13–17^. While this process is still poorly understood, several studies suggest that lung epithelial cells tend to fail acidifying the phagolysosome and by that allow conidia to form hyphae enabling the fungus to disseminate in the body^18,19^. Furthermore, the innate immune system plays an essential role in fungal clearance, comprising various immune effector mechanisms, such as the complement system, the immune response by alveolar macrophages (AM) as resident phagocytes as well as by recruited phagocytes like neutrophils^20–22^. It has been shown that the most efficient killing of *A. fumigatus* conidia is achieved by professional phagocytes, such as AM and that their action prevents fungal persistence and disease development^2,13^. Thus, immune deficiencies associated with these immune effector mechanisms prevent timely clearance of conidia leading to serious infections. Moreover, superinfections caused by fungal pathogens in combination with bacterial or viral pathogens, such as the SARS-CoV-2 virus, worsen the prognosis^23–30^. To improve diagnosis and treatment of these often fatal diseases^23,24,26^, a thorough understanding of the underlying host-pathogen interactions is required.

Observing host–pathogen interactions in the lung in vivo would provide the most realistic insights into spatio-temporal dynamics. However, intravital imaging is hard to accomplish, since it requires advanced techniques in mouse surgery, microscopy and stabilization techniques to overcome respiratory and cardiac contractions^31,32^. Furthermore, it is associated with ethical issues, high costs and is only feasible in mice. An alternative approach is represented by novel organ-on-chip models that allow investigating the host-pathogen interactions in complex physiological microenvironments in vitro and the use of human cell lines^12,33–35^. However, these advanced in vitro models come with limitations, such as being labor and cost intensive as well as limited in the ability to fully represent organ structures like alveoli in all three spatial dimensions^36^.

Here, we follow a systems biology approach by complementing existing experimental studies with computer simulations to address aspects of the host-pathogen interactions that are difficult to access in experiment^37–39^. For a comprehensive overview on mathematical modeling of host-pathogen interactions we refer to Ewald et al.^40^. Briefly, in the context of fungal infections, different techniques have been applied for virtual infection modeling. For example, ordinary and partial differential equations (ODEs/PDEs) have been used to study host-pathogen interactions in well-mixed systems of many particles, both for the fungal yeast *Candida albicans*^41,42^ and the role of AEC during *A. fumigatus* infection^14^. The mathematical concept of game theory was applied to investigate strategies of fungal pathogens interacting with the host allowing to combine model parameters into effective payoffs^43–46^. Furthermore, state-based models (SBM) and agent-based models (ABM) were applied to systems with stochastic and spatial effects in the context of fungal infections^47–55^.

Focusing on infection with *A. fumigatus*, we previously developed three-dimensional to-scale models for the human and murine alveoli in terms of a hybrid agent-based model (hABM)^56–59^, i.e., combining an ABM for simulating cellular interactions with a PDE for simulating molecule secretion, diffusion and uptake. This enabled us to compare infection scenarios in humans and mice for various infection doses ranging from normal daily inhalation up to high doses used in typical mice experiments^58^. Since in vivo experiments can only be done in mice, the question whether these experimental results can be transferred to the infection dynamics in humans can be studied by computer simulations. In addition to the complex interplay between the fungal infection dose and the morphometry of human versus murine alveoli, another essential aspect concerns the unknown number of AM in the lung. Depending on the applied measurement technique as well as the considered patient cohort, a significant range of AM numbers has been reported. While Wallace et al. measured 2.1 · 10^9^ AM in a healthy human lung^60^, Crapo et al. counted more than ten times as many^61^. More recent publications suggest AM numbers around (6–14) · 10^9^ for the healthy human lung^62,63^. In contrast, the measured AM numbers in mice are in the more narrow range of (1.95–3.22) · 10^6^, which is presumably due to higher sample numbers and smaller organ sizes^63–65^.

In this study we investigated the biological question about the quantitative impact of the AM number on the clearance of *A. fumigatus* conidia in humans and mice. To address this question we utilized our previously developed hABM^56–59^ and simulated millions of virtual infection scenarios for various AM numbers and infection doses ranging from daily inhalation to experimentally applied doses. However, numerical hABM simulations are extremely time-consuming (see “Hybrid agent-based modeling of virtual infection scenarios”) limiting efficient analyses and hypothesis testing for the biological systems. Therefore, we derived an analytical surrogate infection model (SIM) that was trained on a large dataset generated by the hABM. The SIM allows to accurately predict outcomes of infection scenarios in a fraction of computation time and thus makes additional time-consuming numerical hABM simulations unnecessary. Moreover, it enables gaining a comprehensive and quantitative understanding of infection dynamics with regard to the comparability of infection clearance for humans and mice under healthy and immunocompromised conditions as well as for various fungal infection doses. In this way, the SIM is capable of predicting minimal AM numbers that are necessary to reach certain threshold values of infection clearance that are measured by an infection score (see “Model input parameter and infection score”).

## Results

**Fig. 1:**
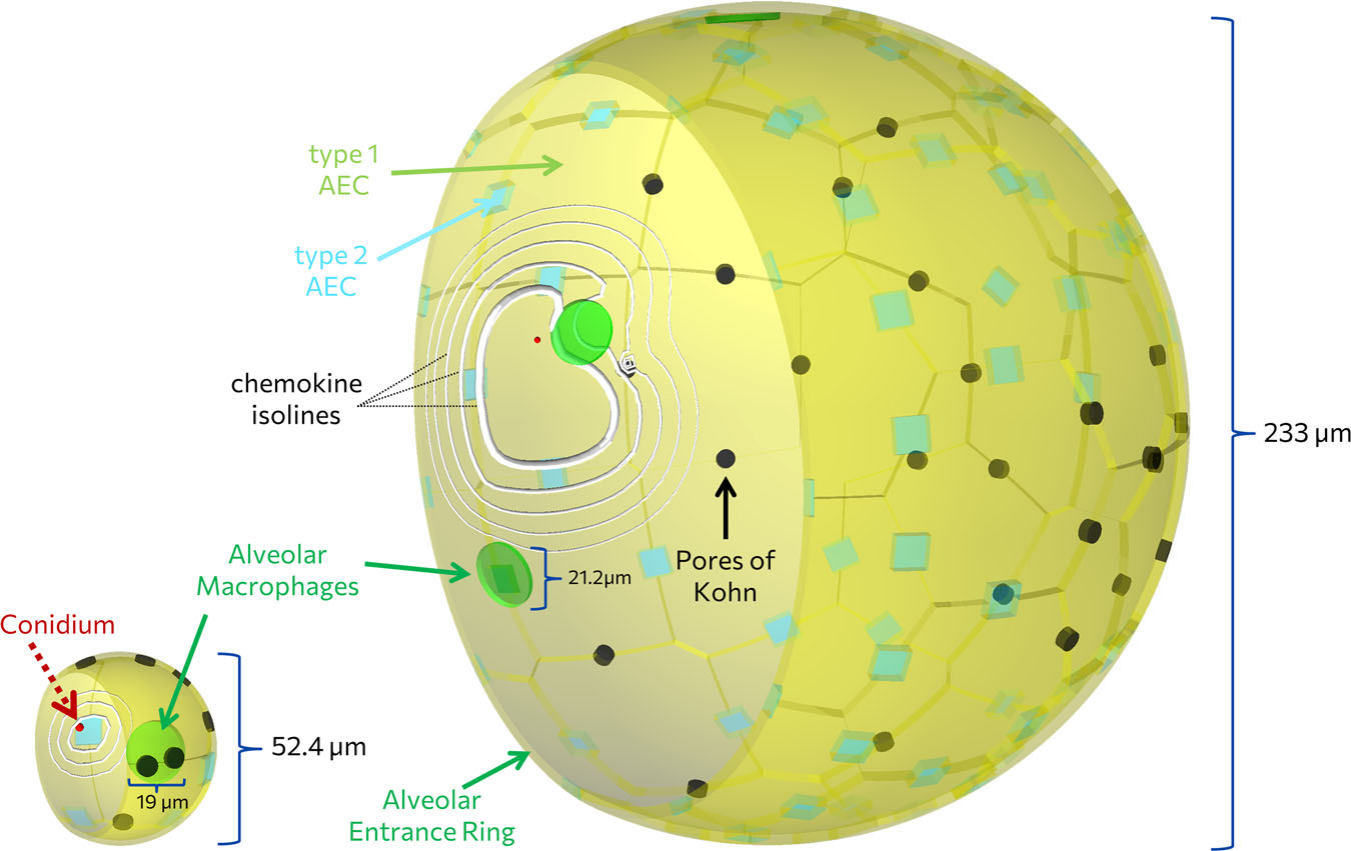
To-scale representation of the murine (left) and human (right) alveolus. The alveolus is modeled as a 3/4 sphere composed of an epithelial cell layer consisting of alveolar epithelial cells (AEC) type I (yellow) and type II (blue). A single *Aspergillus fumigatus* conidium (red) is randomly positioned on the inner alveolar surface and the contacting AEC is secreting chemokines (white isolines) to attract AM (green) towards the conidium. The alveolar entrance ring and the pores of Kohn (black) are the boundaries of the system.

The aim of this study is to quantify *A. fumigatus* infection in human and mouse alveoli, focusing on the effects of the AM number, which has not been clearly determined experimentally to date. Therefore, we calculated the AM and conidia numbers that can be expected in a single alveolus for the human and murine system (see “Model input parameter and infection score” and Table 1). We measure infection clearance by the infection score (*IS*), which corresponds to the probability for the infection to persist a clearance time (*CT*) of 6 h post-infection, i.e., P(*CT* *>* 6*h*) = *IS* (see “Model input parameter and infection score” 1). A visual impression of the to-scale hybrid agent-based model (hABM), as described in “Hybrid agent-based modeling of virtual infection scenarios”, for the human and murine alveolus is given in Fig. 1 and exemplary spatio-temporal computer simulations of various infection scenarios are given by Supplementary Videos 1 and 2. The results of the computationally intensive simulations with the hABM are utilized for the analytical derivation of a Weibull survival model (WSM) valid at a low fungal burden which is extended to a compressed exponential function (CEF) to cover also a high fungal burden. These two approaches were used to derive a surrogate infection model (SIM) for the prediction of infection scores for various input parameters (see “Training a surrogate infection model on hABM simulation data”). A scheme of the entire workflow can be found in Supplementary Fig. 1.

**Table 1:** Summary of screened parameters for the human and murine lung.

Input parameter	Human	Mouse
AM number per alveolus $n_{AM}$	$2,4,6, \ldots ,50$	$0.1,0.2,0.3, \ldots ,2.5$
Number of conidia $n_{Con}$	$1,\;2$	$1,\;2,\;3$
Secretion rate $s_{AEC}$ in $\frac{{molecues}}{{min}}$	$1500,5000,15000,50000,$ $150000,\;500000$	$1500,\;5000,\;15000,\;50000,$ $150000,500000$
Diffusion coefficient $D$ in $\frac{{\mu m^2}}{{min}}$	$20,\;60,\;200,\;600,\;2000,\;6000$	$20,\;60,\;200,\;600,\;2000,\;6000$

For each parameter combination, 5000 simulations were performed. An additional 250 combinations ($s_{AEC} \times D \in \left\{ {1,10} \right\} \times \{ 6000\}$ for all $n_{AM}$ and $n_{Con}$ and both systems) were screened to obtain results for the quasi-random walk (qRW).

### Low fungal burden: Infection score depends exponentially on AM number

**Fig. 2:**
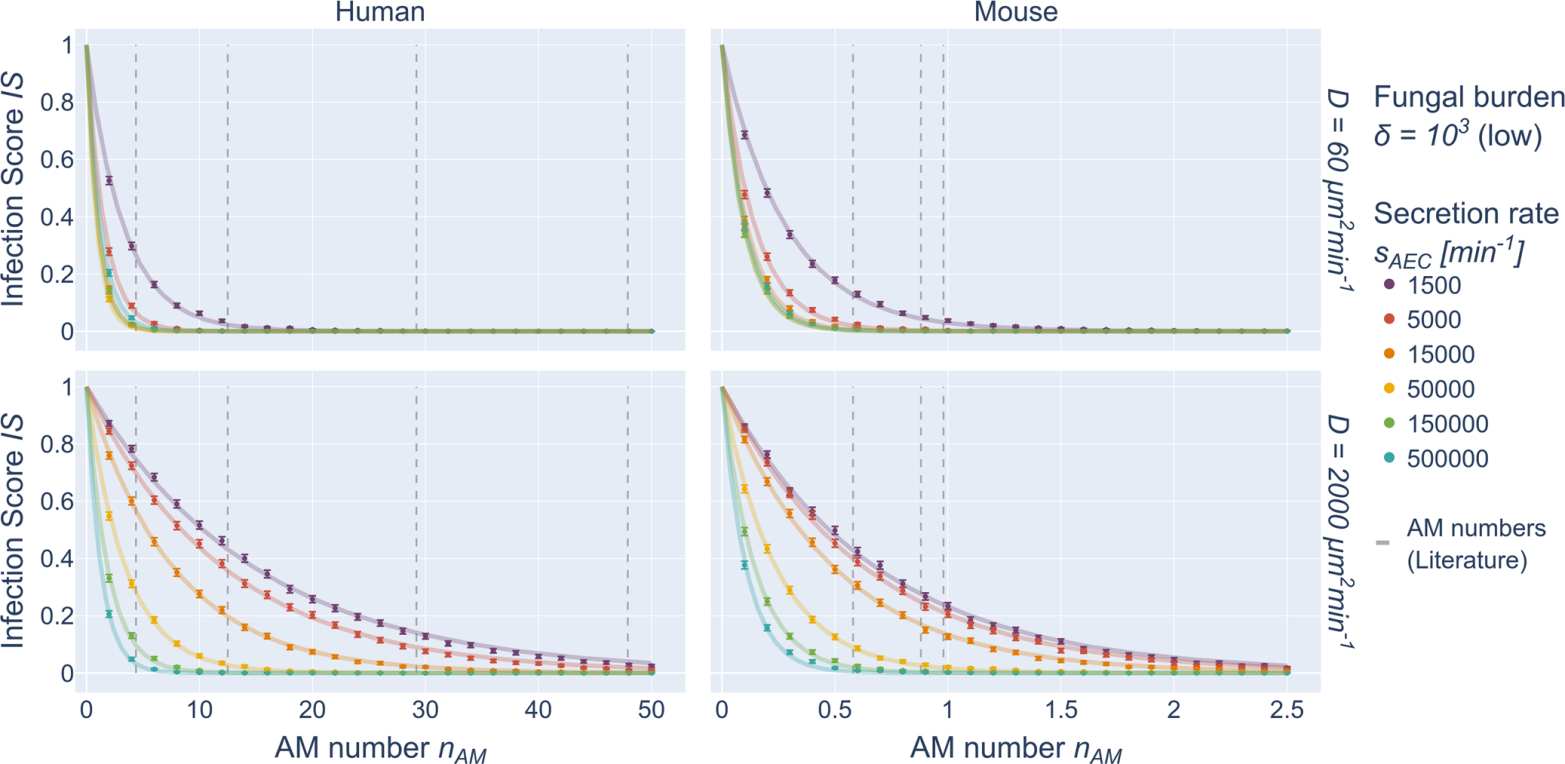
hABM simulation data and SIM predictions for low fungal burden. Simulated data from the hABM are shown as points for low fungal burden in the human (left) and murine (right) system for *D* = 60 µm^2^/min (top row) and *D* = 2000 µm^2^/min (bottom row). Error bars represent the 95% confidence interval as obtained from the standard error of independent Bernoulli trials. Solid lines represent the fit to the data points as obtained by the Weibull survival model (WSM). Dashed vertical lines denote AM numbers from literature.

We first investigated the impact of the AM number on the infection clearance for the case of low fungal burden *δ* = 10^3^, i.e. for an alveolar occupation number (AON) of *n*_*con*_ = 1 conidium per alveolus as the most likely case among all non-empty alveoli (see Supplementary Fig. 2). Computer simulations were performed for increasing AM numbers *n*_*AM*_ in the human and murine alveolus using the hABM to screen the chemokine secretion rate *S*_*AEC*_ and diffusion coefficient *D* (see Table 1). The results in terms of the infection score *IS* are summarized in Fig. 2 and Supplementary Fig. 3. Based on these numerical results we analytically derived a Weibull survival model (WSM):

1 $$IS = P\left( {CT_{s_{AEC},D} > t|n_{AM},n_{Con} = 1} \right) = 1 - \left( {1 - e^{ - \left( {\Lambda t} \right)^Kn_{AM}}} \right)^{n_{Con}} = e^{ - \Lambda ^\prime \;n_{AM}}$$

for $\Lambda ^\prime : = \;\left( {\Lambda t} \right)^K$ and *t* = 6*h* as described in the Supplementary Material section 1.3. The *IS* was computed from 5000 hABM simulations for each parameter configuration $\left( {n_{AM},n_{Con} = 1,s_{AEC},D} \right)$. These data points were fitted by curves that interpolate the *IS* as a function of *n*_*AM*_. The results are depicted as solid lines in Fig. 2 and Supplementary Fig. 3 and match the simulation results with a mean absolute deviation $\left( {MAD_{MODEL}^{s = H,M}} \right)$ of $MAD_{{{{\mathrm{WSM}}}}}^H = 0.0028$ for the human and $MAD_{{{{\mathrm{WSM}}}}}^M = 0.0040$ for the murine system. The *MAD* determines the goodness of fit of a model (here: WSM) to simulated data and corresponds to the fraction of hABM simulation outcomes that are falsely predicted by the model (see “Training a surrogate infection model on hABM simulation data”). This comparison reveals that for low fungal burden the *IS* resembles an exponentially decaying function with increasing AM number *n*_*AM*_.

**Fig. 3:**
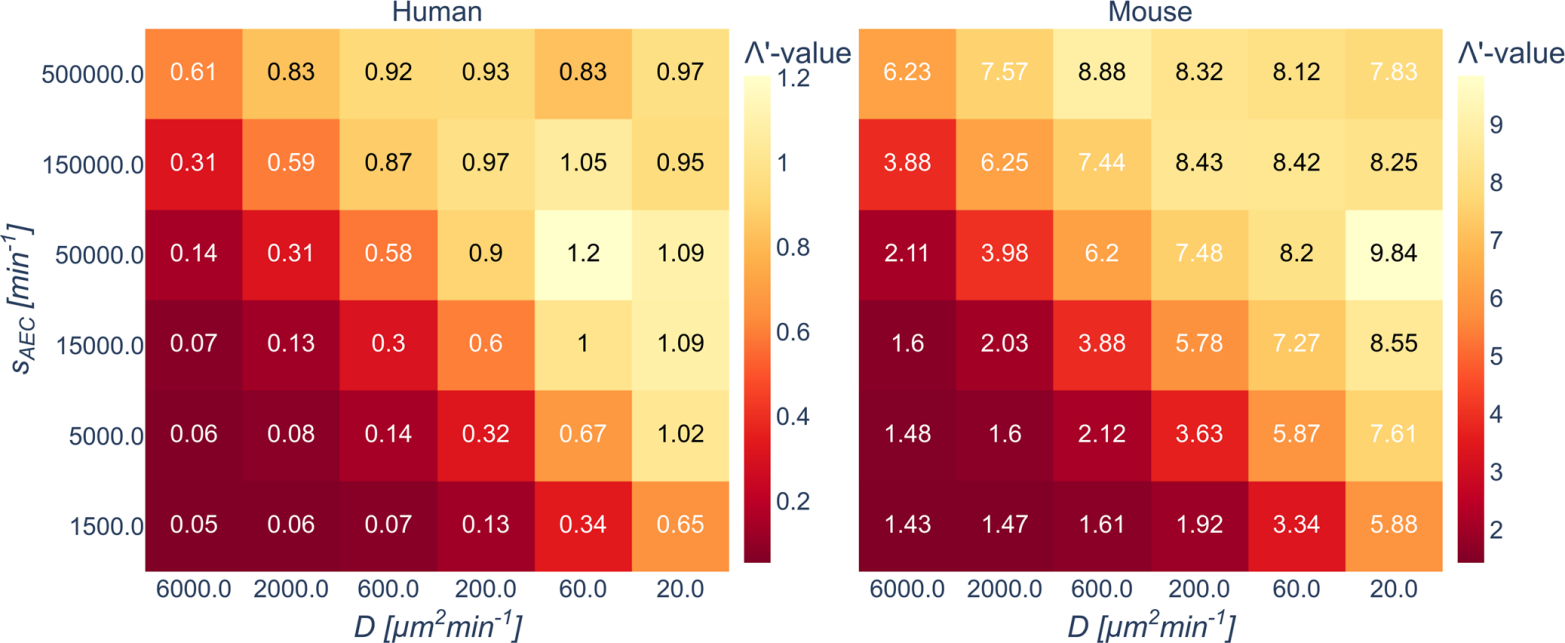
WSM parameters for low fungal burden. Estimated WSM parameters $\Lambda ^\prime$ for low fungal burden and different combinations of $s_{AEC}$ and $D$ in the human and murine alveolus.

By fitting the WSM to the *IS* as obtained from the hABM simulations, we estimated the parameter $\Lambda ^\prime$ for all combinations of secretion rates *S*_*AEC*_ and chemokine diffusion coefficients *D* (see Fig. 3). These estimated $\Lambda ^\prime -$values exhibit a symmetry reflecting the dependence of *IS* on the ratio $s_{AEC}/D$. Computing the mean error $ME_{sym} = \frac{1}{{N^ \ast }}\mathop {\sum}\nolimits_{i,j}^{N^ \ast } {\left| {IS_i^{s = H,M} - IS_j^{s = H,M}} \right|}$ for the *IS* as predicted by hABM simulations for *identical* ratios $\left( {s_{AEC}/D} \right)_i = \left( {s_{AEC}/D} \right)_j$, for values $s_{AEC},D$ taken from Table 1, yielded for both organisms comparably small values of $ME_{sym}^H = 0.0039$ for the human alveolus and $ME_{sym}^M = 0.0059$ for the murine alveolus. This systematic analysis aligns with our previous investigations, which already gave first indications that *IS* correlates with the ratio $s_{AEC}/D$^57–59^. Therefore, in all subsequent investigations we will focus on comparisons for *IS* depending on the ratio $s_{AEC}/D$.

### High fungal burden: Infection score exhibits compressed exponential decrease

**Fig. 4:**
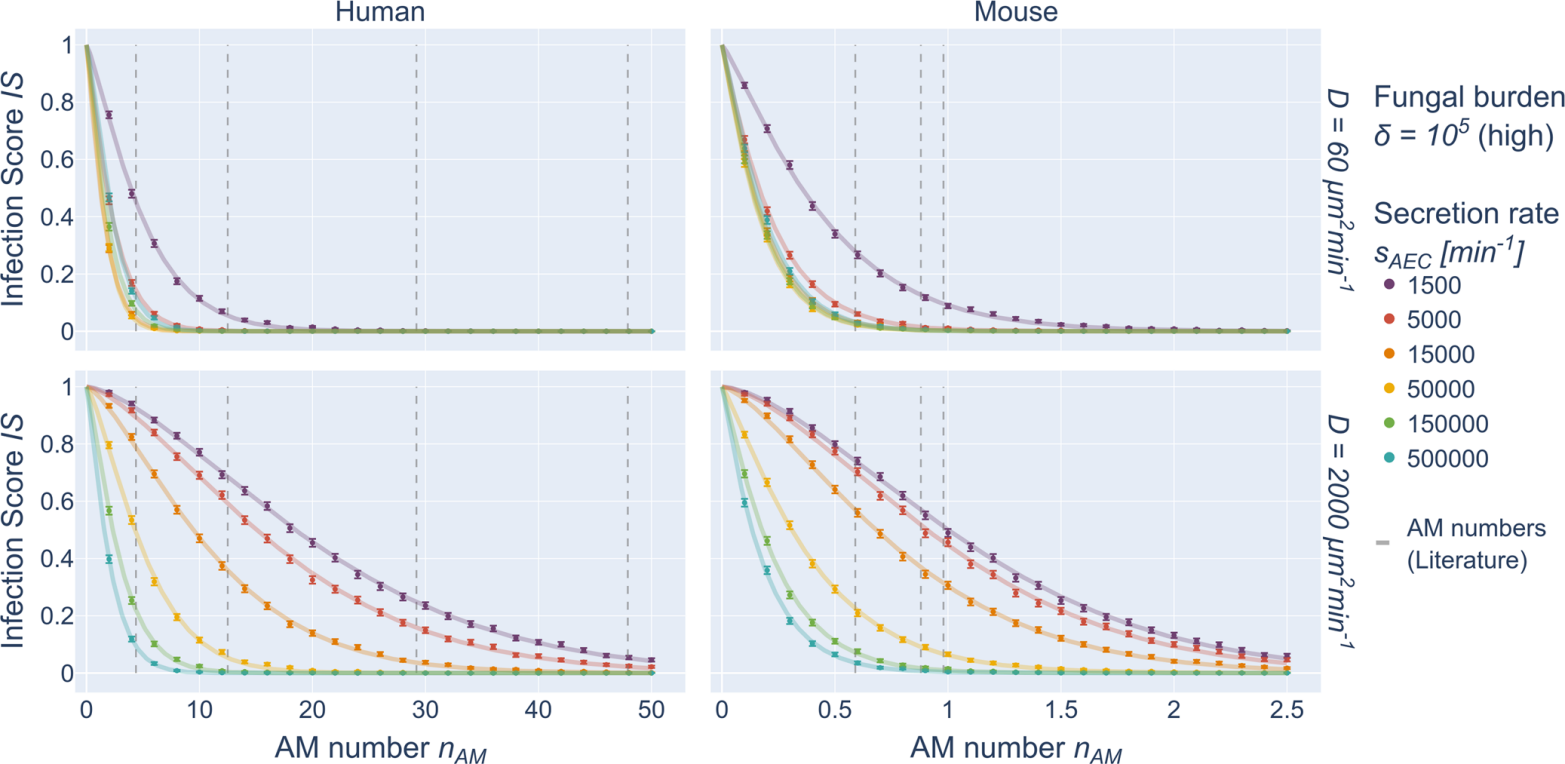
hABM simulation data and SIM predictions for high fungal burden. Simulated data from the hABM are shown as points for high fungal burden in the human (left) and murine (right) system for *D* = 60 µm^2^/min (top row) and *D* = 2000 µm^2^/min (bottom row). Error bars represent the 95% confidence interval as obtained from the standard error of independent Bernoulli trials. Solid lines represent the fit to the data points as obtained by the compressed exponential function (CEF). Dashed vertical lines denote AM numbers from literature.

For an increased fungal burden with $\delta \,>\, 10^3$, multiple conidia can be expected in non-empty alveoli. According to our previous findings^58^, a maximal AON of *n*_*Con*_ = 2 conidia is expected for the human alveolus, even for high fungal burden of $\delta = 10^5$, whereas for the murine alveolus the AON can be as high as *n*_*Con*_ = 3 conidia (see Supplementary Fig. 2). Correspondingly, we performed simulations with the hABM for multiple conidia in the human and murine alveolus varying again the chemokine parameters as well as the AM number. The resulting infection scores are illustrated as colored points in Fig. 4 and Supplementary Fig. 4 and indicate that the *IS* does no longer follow a purely exponential decay in the limit of high fungal burden. Consequently, as indicated in Supplementary Fig. 5, for $MAD_{{{{\mathrm{WSM}}}}}^H = 0.0059$ the previously introduced WSM fits the simulated data in the human system, however in the murine system $\left( {MAD_{{{{\mathrm{WSM}}}}}^M = 0.0201} \right)$ it exceeds the threshold of *MAD* = 0.01 (see “Training a surrogate infection model on hABM simulation data”). Therefore, as described in the “Training a surrogate infection model on hABM simulation data” and as depicted in Supplementary Fig. 6, we re-analyzed the individual Weibull distributed clearance times *CT* assuming a generalized dependence on *n*_*AM*_ and extended the WSM to a compressed exponential function^66^ (CEF),

2 $$P\left( {CT_{n_{Con},s_{AEC},D} > t} \right) \approx e^{ - \beta {n_{AM}}^\gamma},$$

which is capable of capturing deviations from a regular exponential decay of the *IS* in the limit of high fungal burden. The distributions of the fitted CEF parameters *β* and *γ* are shown in Supplementary Fig. 7 illustrating a transition from the exponential behavior of the WSM to the CEF including the limit of low fungal burden for parameters $\beta \approx \Lambda ^\prime$ and $\gamma \approx 1$, for which Eq. (2) reduces to Eq. (1). For the limit of high fungal burden, the fitted CEF for both organisms are shown as solid lines in Fig. 4 and Supplementary Fig. 4 matching the simulation results for various ratios $s_{AEC}/D$ with an $MAD_{{{{\mathrm{CEF}}}}}^H = 0.0021$ for the human and $MAD_{{{{\mathrm{CEF}}}}}^M = 0.0028$ for the murine alveolus.

### Surrogate infection model for prediction of infection scores

A large dataset was generated by simulating more than 23 million infection scenarios by the hABM for the parameter configurations summarized in Table 1. Even though the hABM code is highly optimized, it is associated with demanding computational costs in terms of resources and time (see “Hybrid agent-based modeling of virtual infection scenarios”). To exploit the full potential of this dataset, we trained a surrogate infection model (SIM) that allows making predictions on the infection score *IS* for any configuration of input parameters $p_i^{s = H,M}: = \left( {n_{AM},n_{Con},s_{AEC},D} \right)_i^{s = H,M}$ without having to perform further simulations by the hABM. Motivated by our findings described above regarding the representation of the infection score by the CEF, we made the following attempt for the derivation of the SIM as described in the “Training a surrogate infection model on hABM simulation data”:

3 $${{{\mathrm{SIM}}}}\left( {p;\Theta } \right)^{s = H,M}: = e^{ - f_\beta \left( {n_{Con},s_{AEC},D;\Theta _\beta } \right)\;{n_{AM}}^{f_\gamma \left( {n_{Con},s_{AEC},D;\Theta _\gamma } \right)}}.$$

Here, we introduced generalized functions $f_{x = \beta ,\;\gamma }\left( {n_{Con},s_{AEC},D;\Theta _x} \right)$ that consists of combined logistic functions reflecting the behavior of CEF parameters *β* and *γ* for increasing ratios $s_{AEC}/D$ and varying *n*_*Con*_ (see Supplementary Fig. 7 and “Training a surrogate infection model on hABM simulation data”). Their parameters Θ_*β*_ and Θ_*γ*_ constitute the SIM model parameters Θ = (Θ_*β*_, Θ_*γ*_). SIM(*p*; Θ)^*s*=*H,M*^ was calibrated to the $IS_{p_i}^{s = H,M}$ as obtained from the hABM simulations for all $p_i^{s = H,M}: = \left( {n_{AM},n_{Con},s_{AEC},D} \right)_i^{s = H,M}$ by minimizing the mean squared error *MSE*(Θ) for Θ = (Θ_*β*_, Θ_*γ*_) (see “Training a surrogate infection model on hABM simulation data” and Supplementary Fig. 1). The predictions of the SIM were tested by fitting curves to the data points as shown in Supplementary Figs. 8 and 9 for the human and murine system, respectively. As we reach a $MAD_{{{{\mathrm{SIM}}}}}^H = 0.0051$ for the human and a $MAD_{{{{\mathrm{SIM}}}}}^M = 0.0052$ for the murine system, we conclude that the numerical quality of the prediction of $IS_{p_i}^{s = H,M}$ by the SIM is just as accurate as obtained for the hABM simulations.

**Fig. 5:**
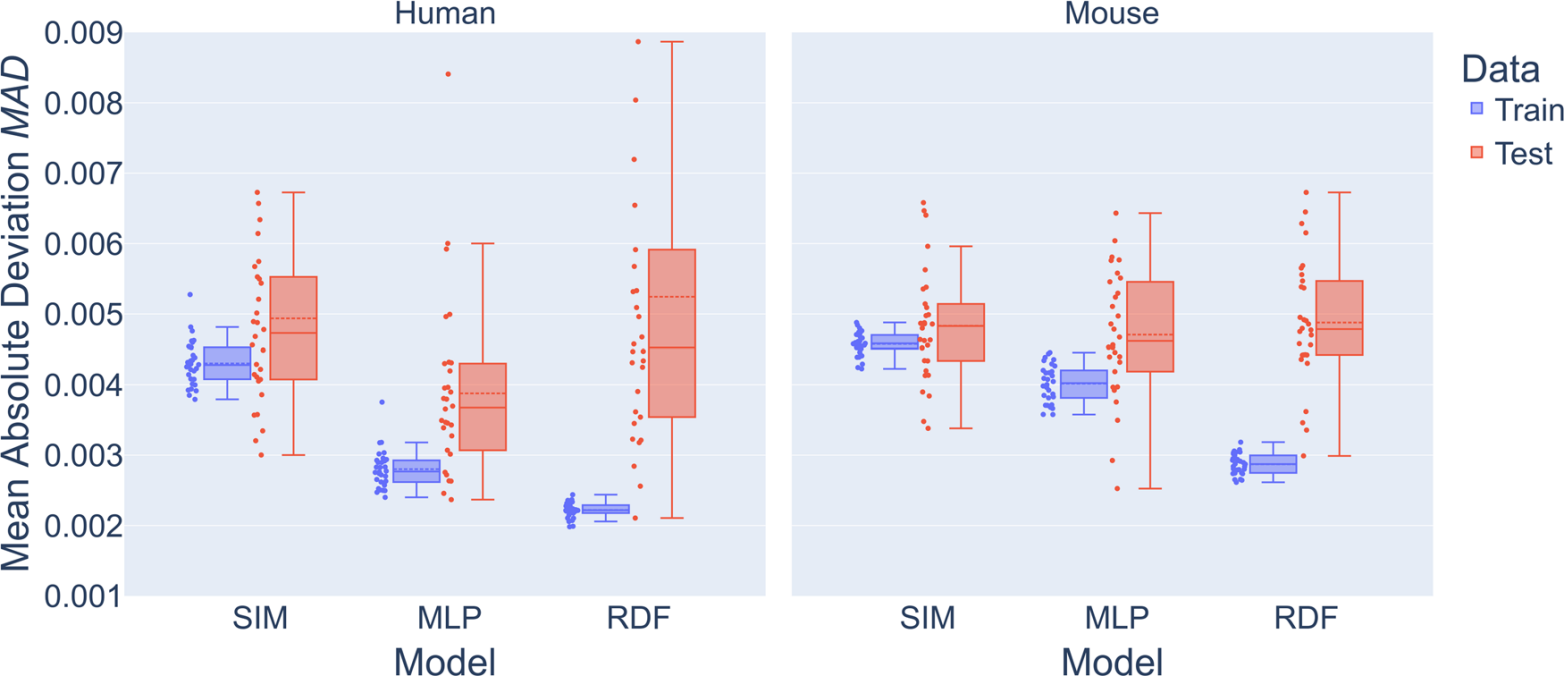
Comparison of SIM performance with MLP and RDF. Mean absolute deviation (MAD) of the surrogate infection model (SIM) with a multilayer perceptron (MLP) and a random decision forest (RDF) via 5 times 6-fold cross-validation with train (blue) and test (red) data. Data points represent the distribution. Boxes represent the top and bottom quartiles of the distribution and whiskers are extended to 1.5 times the interquartile range. Horizontal line represents the median and dashed horizontal line the mean of the distribution.

To further validate our analytically derived SIM, we compared its outcomes with two machine learning approaches that are described in detail in the Supplementary Material section 1.5: a multilayer perceptron (MLP)^67^ belonging to the class of fully-connected feedforward artificial neural networks, and a random decision forest (RDF)^68^ for input parameters $p_i^{s = H,M}$ and output data $IS_{p_i}^{s = H,M}$. Next, for model comparison we performed a five times six-fold cross-validation^69^ (see Supplementary Fig. 1) as obtained from the hABM on all three models: SIM, MLP and RDF. The results are summarized in Fig. 5 showing that the MLP and RDF yielded a slightly lower training error than the SIM. While the test errors were comparable for all three models, the RDF substantially overfits the data, as evidenced by the high deviation between test and training errors compared to the SIM, which exhibits the least tendency to overfitting. The accuracy of the SIM is comparable to the machine learning models, and it reveals hidden system dynamics requiring seven times less parameters. In what follows, we apply the SIM to quantitatively analyze the impact of the AM number on the *IS* to compare infection clearance in the human and murine system.

### SIM predicts optimal chemokine parameters minimizing the infection score

**Fig. 6:**
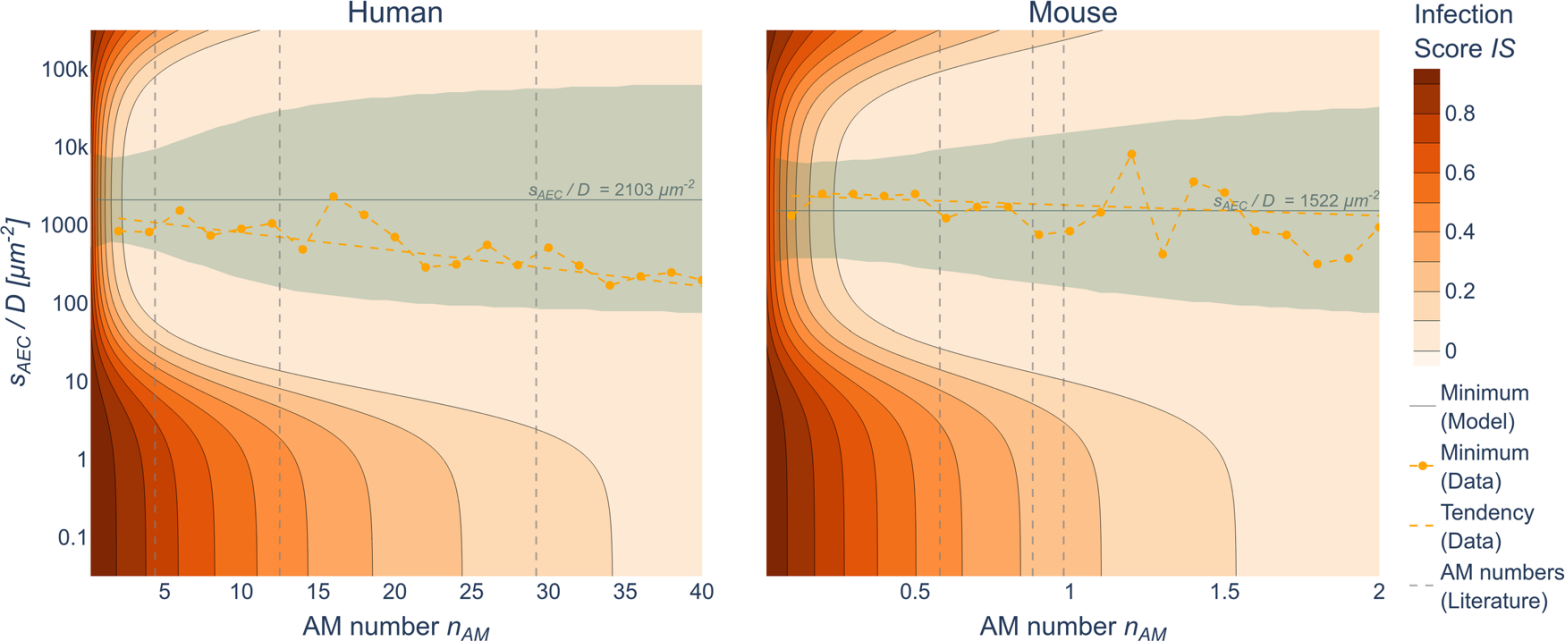
SIM predictions of optimal chemokine ratios. Predicted infection scores (orange gradient) by the surrogate infection model (SIM) for pairs of AM numbers $n_{AM}$ and chemokine ratios $s_{AEC}/D$ for low fungal burden (see Supplementary Fig. 10 for high fungal burden) in the human (left) and murine (right) alveolus. Dashed vertical lines denote AM numbers from literature. The gray area represents the 95% confidence interval around optimal value $s_{AEC}/D$ for the lowest infection score derived from the SIM. Yellow points represent optimal values derived from the simulation data (yellow dashed lines as a guide for the eye).

Using the SIM we were able to obtain the optimal ratio $s_{AEC}/D$ of the chemokine parameters by minimizing *IS* for a given AM number. As can be seen in Fig. 6, for chemokine ratios in the range $10^2{{{\mathrm{\mu }}}}m^{ - 2} \,<\, s_{AEC}/D \,<\, 5 \cdot 10^4{{{\mathrm{\mu }}}}m^{ - 2}$ in the human and $5 \cdot 10^2{{{\mathrm{\mu }}}}m^{ - 2} \,<\, s_{AEC}/D \,<\, 2 \cdot 10^4{{{\mathrm{\mu }}}}m^{ - 2}$ in the murine system, there exist large regions with *IS* < 0.1. This is the case for $n_{AM} \,>\, 2$ in the human system and $n_{AM} \,>\, 0.3$ in the murine system for low fungal burden with $\delta = 10^3$. For high fungal burden with $\delta = 10^5$ these values increased to $n_{AM} \,>\, 4.4$ in the human and $n_{AM} \,>\, 0.45$ in the murine system (see Supplementary Fig. 10). It can also be seen in Fig. 6 (Supplementary Fig. 10) that the minimal *IS* was reached for the chemokine ratio $s_{AEC}/D = 2103\;\mu m^{ - 2}$($s_{AEC}/D = 2103\;\mu m^{ - 2}$) for the human and $s_{AEC}/D = 1522\;\mu m^{ - 2}$ ($s_{AEC}/D = 1606\;\mu m^{ - 2}$) for the murine system at low (high) fungal burden. The gray area denotes all chemokine ratios $s_{AEC}/D$ where the *IS* deviates less than 5% from the optimal ratio for each AM number.

To compare the SIM with the hABM, we computed the weighted minimum^58^ of the chemokine ratios $s_{AEC}/D$ for all hABM simulations with *IS* value within the 95% confidence interval of the optimal chemokine ratio $s_{AEC}/D$ (see Supplementary Material section 1.6). These values are depicted as the orange dashed line in Fig. 6 and Supplementary Fig. 10 and are within the 95% confidence interval of the SIM for both systems. On average the optimal chemokine ratios calculated from hABM simulations were lower than the predicted optimal chemokine ratios from the SIM for the human system for low as well as high fungal burden and exhibited a decreasing trend for increasing AM numbers. For the murine system, the calculated optimal chemokine ratios were very similar to the predicted values from the SIM for low as well as high fungal burden.

### SIM predicts minimal AM number to reach targeted infection score

**Fig. 7:**
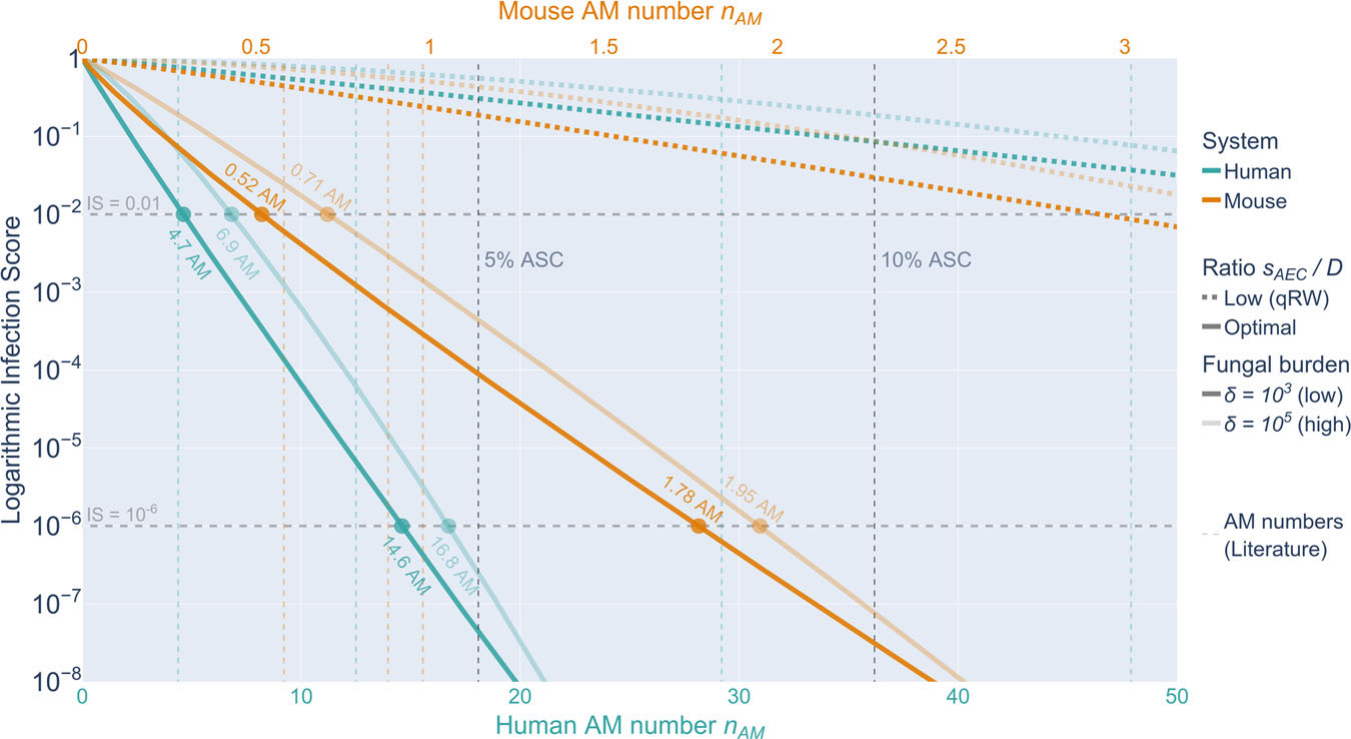
SIM predictions of infection scores. Log-scaled infection score for quasi-random walk qRW (dotted curves) and optimal ratio $s_{AEC}/D$ (solid curves) in the case of low (dark colors) and high (bright colors) fungal burden in the human (green) and murine (orange) alveolus. Colored dashed vertical lines represent the corresponding AM numbers as obtained by literature data. Black dashed vertical lines denote the 5% and 10% ASC for both organisms.

The analytical representation of the infection score *IS* by the SIM enabled estimating a lower bound of the AM number $n_{AM}$ that is required to reach a certain *IS*. This is shown in Fig. 7 with the absolute AM number $n_{AM}$ normalized to the organism’s alveolar surface area, i.e. the percentage alveolar surface coverage (ASC). For both, the optimal chemotactic migration as well the quasi-random walk (qRW), where almost no chemokine signal is present, a superlinear relationship was observed between the log-scaled *IS* and the increasing AM number. For optimal chemokine parameters shown with solid lines, the slope in the range between $10^{ - 2}$ and $10^{ - 6}$ of the log-scaled *IS* over the AM number showed that by adding on average 2.5 AM ($ASC = 0.69\%$) to the human or 0.31 AM ($ASC = 1.4\%$) to the murine alveolus decreases the infection score *IS* 10-fold in the limit of both low and high fungal burden. Furthermore, as can be seen in Fig. 7 and Supplementary Table 1, a minimum of 4.7 AM per alveolus, corresponding to an $ASC = 1.3\%$, is required to achieve an infection score $IS \,<\, 0.01$ in the human system; thus, the probability for persistence of undetected conidia after 6 hours is less than 1%. Accordingly, in the murine system the minimum AM number is 0.52 corresponding to an $ASC = 2.3\%$. For high fungal burden, at least 6.9 AM ($ASC = 1.9\%$) and 0.71 AM ($ASC = 3.1\%$), respectively, in the human and murine system are required to reach an infection score $IS \,<\, 0.01$.

As shown by the green and orange dotted curves in Fig. 7, the chemokine ratio $s_{AEC}/D$ for a qRW was predicted to be extremely low corresponding to the limit of absent chemokine signaling, where the immune defense does not reach a limit of $IS \,<\, 0.01$ for less than 65 AM ($ASC = 18\%$) in the human and 2.9 AM ($ASC = 13\%$) in the murine system. These numbers increased up to 70 AM ($ASC = 19\%$) and 3.4 AM ($ASC = 14\%$), respectively, for humans and mice in the case of a qRW for high fungal burden.

### SIM compares infection scores in humans and mice for arbitrary chemokine ratios

The SIM allows continuous representations of infection scores for the human and murine system for any values within the ranges of the scanned input parameters and thus enabled the comparison of both organisms. The corresponding *IS* for low and high fungal burden is shown in Supplementary Video 3 for varying chemokine parameters from the qRW to the optimal chemokine ratio $s_{AEC}/D$. In the case of a qRW the *IS* is higher in the human alveolus for an equal ASC. As can be expected, for an increasing ratio $s_{AEC}/D$ the *IS* decreases for both organisms. Interestingly, for a chemokine ratio $s_{AEC}/D \,>\, 10\;\mu {\rm{m}}^{ - 2}$ ($s_{AEC}/D \,>\, 3\;\mu {\rm{m}}^{ - 2}$) for low (high) fungal burden, we observed a switch in the *IS*, i.e. for equal ASC the infection score becomes smaller in the human system compared to the murine system.

**Fig. 8:**
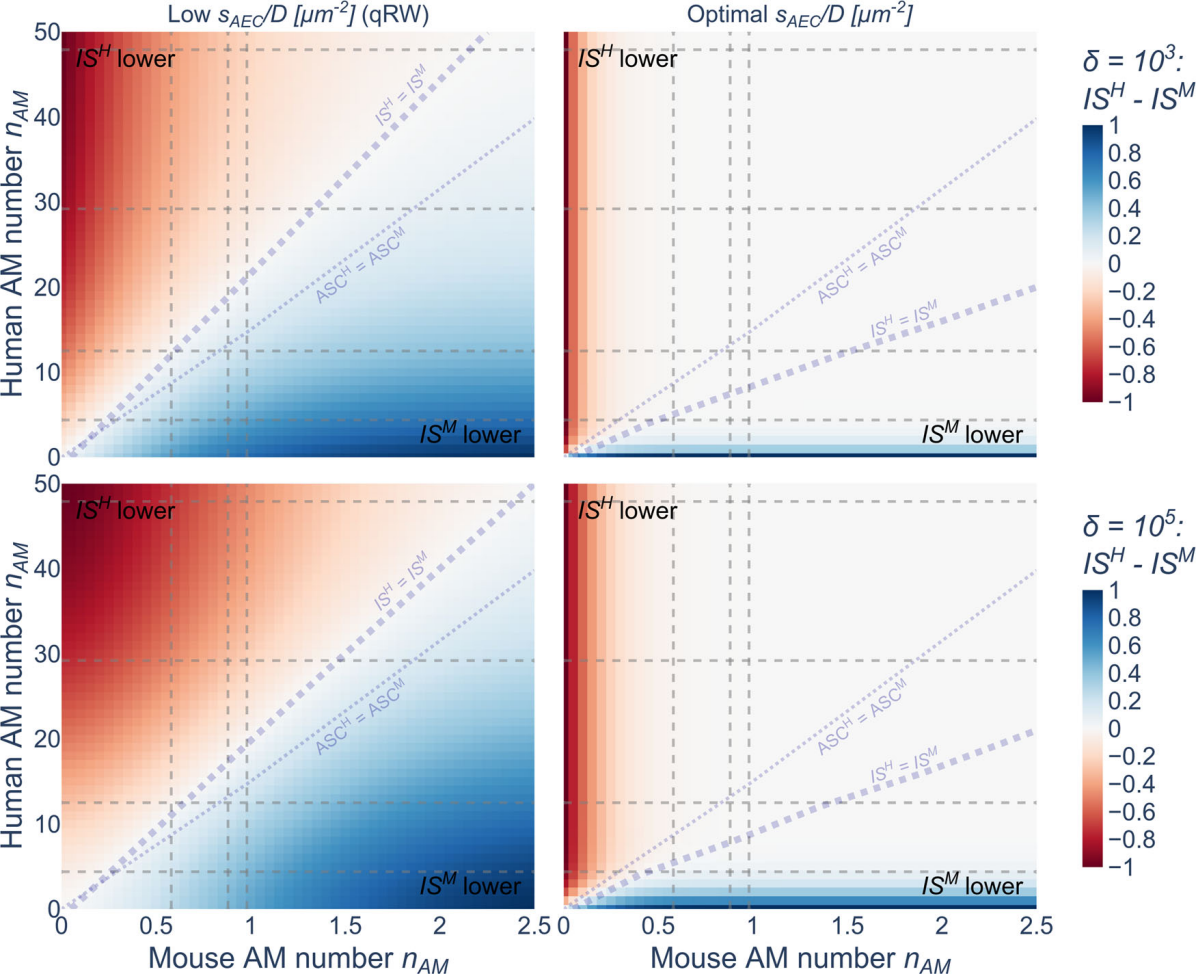
Comparison of human and murine infection scores. Cross-comparison of infection scores between AM numbers $n_{AM}$ in the human (Y-Axis) and murine (X-Axis) system including literature values for human (dashed horizontal lines) and mouse (dashed vertical lines). Red areas denote $IS^H \,<\, IS^M$, white areas show where $IS^H \approx IS^M$ and blue areas show where $IS^H \,>\, IS^M$. The diagonal dotted fat gray line denotes the limit where both systems have an equal IS ($IS^H = IS^M$). The diagonal dotted gray line denotes an equal ASC for both organisms.

In addition to comparing both systems for equal ASC, we performed a cross-comparison of the infection score *IS* for different AM numbers in both organisms for qRW and optimal ratio $s_{AEC}/D$ as well as for low and high fungal burden. In Fig. 8, three distinct regimes can be identified for both modes of AM migration: (i) a regime where the *IS* is lower in the murine system (blue) (ii) a regime where the *IS* is lower in the human system (red) and (iii) a regime where both organisms have an equal *IS* (white). In all cases, the colored upper-left and bottom-right regimes denote that both systems have a lower *IS* when they have many AM compared to very few AM in the other system. For the qRW migration, we only found a relatively narrow region along the diagonal where both organisms performed equally well, whereas, for optimal chemokine ratios, this is the case for the majority of combinations denoted by the white area.

Furthermore, we performed this cross-comparison with regard to the upper boundaries of the literature values for the human $\left( {n_{AM}^H \le 47.9} \right)$ and murine system $\left( {n_{AM}^M \le 0.98} \right)$. In case of the qRW, the human system would require $n_{AM}^H \ge 21.7$ for a low and $n_{AM}^H \ge 19.8$ for high fungal burden to reach *IS* equal or lower as the murine system, i.e. $IS^H \le IS^M$. In contrast, the murine system would require $n_{AM}^M \ge 2.2$ for a low and $n_{AM}^M \ge 2.4$ for high fungal burden to reach *IS* equally or lower as the human system, i.e. $IS^M \le IS^H$. For both – low and high fungal burden – the qRW under the condition $IS^H = IS^M$ is associated with $n_{AM}$ values being always above the curve at which $ASC^H = ASC^M$. This indicates that for qRW the murine system can clear infections more efficiently for equal ASC.

For optimal chemokine ratios, the human system would require $n_{AM}^H \ge 8.4$ for a low and $n_{AM}^H \ge 9.1$ for high fungal burden to reach $IS^H \le IS^M$, while the murine system would require $n_{AM}^M \ge 6.4$ for a low and $n_{AM}^M \ge 6.4$ for high fungal burden to reach $IS^M \le IS^H$. Interestingly, the curve $IS^H = IS^M$ lies below the curve at which $ASC^H = ACS^M$ for optimal chemokine ratios, indicating that in this case the human systems is more efficiently clearing infections compared to the murine system.

## Discussion

In this study, we investigated the impact of the number of AM—the resident immune cells in the lung - on the infection clearance of the opportunistic fungus *Aspergillus fumigatus*. The AM number remains controversial with measurements ranging from (2.1–23) · 10^9^ AM in the human lung. We here utilized previously developed hybrid agent-based models of the human and murine alveolus^56–59^. In addition to the AM number, we screened the fungal burden, which is the expected number of conidia per alveolus, as well as chemokine parameters in terms of the secretion rate and diffusion coefficient referring to the chemotactic signals generated by alveolar epithelial cells to direct AM migration. Thus, we simulated millions (see “Hybrid agent-based modeling of virtual infection scenarios”) of virtual infection scenarios and based on the time-consuming generation of this large dataset we developed a surrogate infection model (SIM) that allows fast and accurate prediction of infection scores for various conditions in the human and murine system.

First, we were able to show that in the limit of low fungal burden, the infection score *IS* decreases exponentially for increasing AM numbers and that the individual clearance times *CT* follow a Weibull distribution for each parameter configuration (see Fig. 2 and Supplementary Fig. 3). The well-known Weibull survival model (WSM) is commonly used to compute the reliability of parallel systems^70,71^ in terms of failure times assuming independence of the random variables. In the present context, where the random variables correspond to the *CT* of each AM, we can conclude that multiple AM within a single alveolus do not seem to be correlated in the limit of low fungal burden. The estimated WSM parameters were found to be symmetric in the chemokine parameters $s_{AEC}$ and *D* for both low and high fungal burden (see Fig. 3). Thus, we concluded that the infection clearance remains the same if the amount of secreting of molecules $s_{AEC}$ and the diffusion coefficient *D* are scaled equally. Therefore, we based our further investigations on screening the chemokine ratio $s_{AEC}/D$, which is a quantity directly related to the chemokine gradient at the chemokine source^72^, i.e. the border of the conidia-associated alveolar epithelial cell.

Next, we investigated infection dynamics for high fungal burden, i.e., with an AON of $n_{Con} = 2$ in the human alveolus and $n_{Con} = 2$ or $n_{Con} = 3$ in the murine alveolus. Here, the previously used WSM did not fit the hABM data for all conditions as it can be seen in Supplementary Fig. 5. In particular, the murine system was affected, which indicates a violation of the independence criteria of the WSM in the presence of multiple chemokine sources (see technical derivation in the Supplementary Material section 1.3). This implies that AM migration was substantially influenced by multiple secreting alveolar epithelial cells in the limit $n_{con} \,>\, 1$. Surprisingly, in the murine alveolus, the computed *IS* based on the hABM simulations yielded values that were lower compared to those predicted by the WSM. In other words, clearing times of AM were positively affected in the presence of more than one targeted conidium. As this effect was not observed for the human alveolus in the case of high fungal burden, we conclude that this observation is due to its substantially larger surface area compared to the murine alveolus, which implies that the search by AM in the human alveolus can be viewed as independent events even for high fungal burden. Thus, in contrast to the human alveolus, AM in the murine alveolus receive chemokine signals continuously, such that their motion remains uninterruptedly in the mode of optimal chemotactic migration from one conidium to the next. Detailed simulations accompanied with a correlation analysis of AM tracks may be performed in the future to investigate this effect in full detail.

In order to accurately fit the simulation results of the hABM, we extended the WSM to a compressed exponential function^66^ (CEF), which has one additional parameter. The resulting fits are shown in Fig. 4 and Supplementary Fig. 4 for low and high fungal burden, where the estimated CEF parameters *β* and *γ* followed a logistic growth curved with a logistic decline along the ratio $s_{AEC}/D$ and were shifted for different AON of $n_{Con} \in \{ 1,2,3\}$ (see Supplementary Figs. 6, 7). Based on these dependencies we derived the surrogate infection model (SIM) that approximates the CEF parameters by the functions $f_\beta ,f_\gamma$. Fitting the SIM to the hABM data accurately reproduced the *IS* data as previously obtained by the hABM (see Supplementary Figs. 8, 9). The performance of our SIM was comparable to state-of-the-art machine learning models (see the result of k-fold cross-validation in Fig. 5) with the advantage of requiring 7-times fewer model parameters. In contrast to these black-box models, the construction and analytical derivation of the SIM not only reduces time-consuming numerical simulations by the hABM, but this white-box modeling approach also provides interpretable results on the host-pathogen interactions, which are discussed below.

We used the SIM to predict optimal chemokine ratios $s_{AEC}/D$ for low and high fungal burden in both systems (see Fig. 6 and Supplementary Fig. 10) and found that the human system showed a 38% (31%) increased optimal ratio $s_{AEC}/D$ in the case of low (high) fungal burden compared to the murine system. We conclude that this increase in the murine system is the consequence of an almost 20-times larger alveolar surface area in the human alveolus, which may also give rise to a wider confidence interval reflecting greater variability. Interestingly, the optimal ratio $s_{AEC}/D$ increased slightly for high fungal burden in the murine system, whereas it remained the same in the human system. In comparison, the optimal values for $s_{AEC}/D$ found by Blickensdorf et al.^58^ $\left( {s_{AEC}/D^H = 423\;\mu {\rm{m}}^{ - 2},s_{AEC}/D^M = 1081\;\mu {\rm{m}}^{ - 2}} \right)$ based on $n_{AM}^H = 4.38$ and $n_{AM}^M = 0.72$ are within the SIM provided 95% confidence interval. For these particular AM numbers, our simulations confirm the earlier findings by Blickensdorf et al.^58^. Interestingly, averaging over the AM numbers for each organism (Table 1), the SIM predicted that the optimal chemokine ratio for humans is higher than for mice. In particular, the optimal chemokine ratio can be expected in the range $4 \cdot 10^2\mu {\rm{m}}^{ - 2} \,<\, s_{AEC}/D \,<\, 10^4\mu {\rm{m}}^{ - 2}$ for low AM numbers ($ASC^{S = H,M} \,<\, 3\%$) and in the extended range $10^2\mu {\rm{m}}^{ - 2} \,<\, s_{AEC}/D \,<\, 5 \cdot 10^4\mu {\rm{m}}^{ - 2}$ for high AM numbers $\left( {ASC^H \,>\, 5\% ,ASC^M \,>\, 10\% } \right)$. For chemokine ratios $s_{AEC}/D \,<\, 10^2\mu {\rm{m}}^{ - 2}$ (see Fig. 6) either too few molecules are secreted by the AEC or the secreted molecules diffuse quickly, which both will be associated with a too weak chemokine signal for efficient guidance of AM to the sites of infection and a relatively high infection score *IS*. In contrast, for chemokine ratios $s_{AEC}/D \,>\, 5 \cdot 10^4\mu {\rm{m}}^{ - 2}$ (see Fig. 6) either too many molecules are secreted by the AEC or the molecules diffuse too slowly, such that chemokines accumulate around the source of infection without any long-range effect on AM recruitment, which is associated with a relatively high value of the infection score *IS*.

To estimate realistic AM numbers in the human system, we consider the narrow range of AM numbers $n_{AM}^M \in \left[ {0.59,0.98} \right]$ for the murine system as this is based on high sample numbers given in the literature^63–65^. Assuming a comparable percentage of alveolar surface coverage (ASC) in both systems would translate the AM number in the human system to the range $n_{AM}^H \in \left[ {9.4,15.6} \right]$. Additionally, based on the identified optimal chemokine ratios we could also utilize the SIM to predict the minimal AM number required for achieving specific infection scores *IS* (see Fig. 7, Supplementary Table 1). The experimentally obtained lower limit of the AM number of $n_{AM}^M = 0.59$ in the murine alveolus yielded an infection score $IS = 5 \cdot 10^{ - 3}$ for low fungal burden. If we require this infection score to set the limit in the human system, we obtain the AM number $n_{AM}^H = 5.3$. In the limit of high fungal burden, translating $IS = 0.024$ for the minimum AM number in the murine to the human system, yields a minimum AM number of $n_{AM}^H = 5.8$ in the human system as predicted by the SIM. These numbers indicate that the experimentally determined AM number of $n_{AM}^H = 4.38$, as provided in the study by Wallace et al.^60^, is quantitatively just a bit lower than the AM number that would be expected by a direct translation from the murine to the human system. On the other hand, the experimentally determined higher AM numbers $n_{AM}^H = 47.9$ $\left( {n_{AM}^H = 29.2} \right)$, as reported by Crapo et al.^61^ *(*Hume et al.^62^*)*, would correspond to an ASC > 13% (ASC > 8%) and would be associated with an extremely low infection score $IS \,<\, 10^{ - 21}$ ($IS \,<\, 10^{ - 12}$) as predicted by the SIM. Assuming the immune defense to operate optimally, including efficient resource management by the organism, such AM numbers may be considered unexpectedly high for a healthy human lung. In fact, we suggest that elevated AM numbers at such high levels may rather be an indicator for dysfunctional chemokine signaling in patients, comorbidity or the side effect of some treatment. Based on our analysis, we arrive at the conclusion that an AM number in the range $n_{AM}^H = 12.5$ as measured by Stone et al.^63^ and associated with infection score $IS \,<\, 10^{ - 6}$ may represent a realistic value for the healthy human lung.

In conclusion, the newly developed SIM captures hABM simulation results and enables us to predict infection scores for various infection scenarios. Our models may be extended to also include predictions on the efficacy of drug treatment and the infection dynamics of other pathogens as well as immunodeficiencies. Further in vitro and in vivo experiments are necessary to (i) validate our quantitative model predictions and (ii) narrow down scanned parameter ranges in order to reduce computational complexity. In particular, animal models as well as innovative methods based on the organ-on-chip technology may provide necessary experimental data for enhancing and refining the hybrid agent-based modeling approach. These would allow to include more detailed, multifactorial effects such as phagocytic behavior of alveolar epithelial cells^15–19^ or the role of neutrophils^20–22,44^. Also, more complex environmental structures could be considered, i.e., a model extension to simulate recruitment of immune cells from alveolus-surrounding capillaries or from the alveolar duct, to make the model as realistic as possible and to fully exploit the potential of computational biology.

## Methods

In this study we compared the infection dynamics of *A. fumigatus* for varying numbers of AM that have been reported for the human^60–63^ and murine^63–65^ lung. Computer simulations were performed for various infection doses as well as different chemokine diffusion conditions. The following subsections provide a detailed overview of the model input parameters and the quantification of the model output as well as a description of the hybrid agent-based model (hABM) for simulating the virtual infection scenarios. Moreover, we introduce a newly developed surrogate infection model (SIM) based on a large database of millions of numerical simulations by the hABM.

### Model input parameter and infection score

In the literature various measures for the AM number in the human lung are reported ranging from $(2.1 - 23) \cdot 10^9$ ^60–63^. Assuming that AM are uniformly distributed over all alveoli, we calculated a range of $n_{AM}^H \in \left[ {4.38,47.9} \right]$ AM per alveolus for a total number of about $n_{alv}^H \approx 4.8 \cdot 10^8$ alveoli in the human lung^73^. Similarly, we identified the range of $n_{AM}^M \in \left[ {0.59,0.98} \right]$ AM given the much lower number of alveoli $n_{alv}^M \approx 3.3 \pm 1.3 \cdot 10^6$ in mice. Note that $n_{AM}^{s = H,M}$(with $s = H$ for the human and $s = M$ for the murine system) represents the time-averaged number of AM leaving and entering an alveolus.

Similarly, we calculated the expected number of conidia $n_{Con}$ per alveolus, which depends on the infection dose that is inhaled by the organism. In our simulations we considered infection doses ranging from normal daily inhalation by humans of about $10^3$ conidia up to relatively high doses administered in typical mice experiments of about $10^6\!\!-\!\!10^8$ conidia. The amount of these conidia that actually reaches the lower respiratory tract is referred to as the fungal burden *δ* and is found to be in the range of $10^3\!\! -\!\! 10^5$ conidia in the murine lung^74,75^. The alveolar occupation number (AON) can be derived from the binomial distribution^58^: For low fungal burden corresponding to the dose of daily inhalation, $\delta = 10^3$ conidia, a non-empty alveolus with one conidium, $n_{Con}^{s = H,M} = 1$, is the most probable case for both organisms. For a high fungal burden of up to 10^5^ conidia the AON rises to $n_{Con}^M = 3$ conidia in the murine system, while this number will not exceed $n_{Con}^H = 2$ conidia in the human system (see Supplementary Fig. 2)^58^. Hence, we simulated infection scenarios for AON with $n_{Con}^H \in \{ 1,2\}$ for the human system and $n_{Con}^M \in \{ 1,2,3\}$ for the murine system.

In the hABM, we model the secretion of chemokines by AEC that harbor a conidium to attract AM, e.g. by the macrophage inflammatory protein-2 (MIP-2)^76^, the monocyte chemoattractant protein (MCP-1)^77^ or the granulocyte macrophage colony stimulating factor (GM-CSF)^78^. For both systems and each combination of $\left( {n_{AM},n_{Con}} \right)$, we screened the chemokine secretion rate $s_{AEC}$ and the diffusion coefficient *D* in experimentally relevant ranges^57–59^. This results in parameter configurations $p_i^{s = H,M}: = \left( {n_{AM},n_{Con},s_{AEC},D} \right)_i^{s = H,M}$ with $i \in \left\{ {1, \ldots ,n^{\left. {s = H,M} \right)}} \right\}$, where $n^{s = H,M}$ denotes the total number of parameter configurations. As can be seen in Table 1, the total number of parameter configurations for the human and murine system, respectively, is $n^H = 1900$ and $n^M = 2850$. Each parameter configuration was repeatedly simulated 5000 times yielding in total more than 23 million simulations for both systems. Each simulation was performed until the maximal simulation time $t_{max} = 740\;{{{\mathrm{min}}}}$ was reached with a time step $\Delta t \le 0.1\;min$, where higher diffusion coefficients *D* require lower time steps $\Delta t$ to guarantee computational stability^57^.

In order to quantify infection clearance we apply the previously defined infection score (*IS*) for any input configuration and for both organisms in the same way as^56–59^:

4 $$IS = P\left( {CT \,>\, 6h} \right),$$

where *P* is a probability function and *CT* denotes the clearance time. The *CT* corresponds to the accumulated first-passage times for all conidia in the alveolus, i.e. the duration until the last conidium was encountered by an AM. Since *A. fumigatus* conidia in the alveolus are likely to grow hyphae around six hours post infection^7,12^, we consider this time point to be critical for infection clearance. The *IS* is computed as the fraction of simulations in which not all conidia are detected by AM within the first six hours post infection. Moreover, the high number of simulations of 5000 per parameter configuration guarantees a small 95% confidence interval of $CI = 1.96 \cdot \sqrt {IS\left( {1 - IS} \right)/5000}$ < 0.015 per estimated $IS \in [0,1]$.

### Hybrid agent-based modeling of virtual infection scenarios

We utilized our previously developed spatio-temporal hybrid agent-based model (hABM)^56–59^. This computational framework (see Code availability) allows to simulate virtual infection scenarios for *A. fumigatus* conidia in a single alveolus and to compare the infection clearance for various conditions by the infection score (see Fig. 1 and Supplementary Videos 1 and 2).

The hABM comprises a realistic to-scale representation for both the human and murine alveolus consisting of a 3⁄4 sphere with AEC of type 1 and 2 as well as pores of Kohn (PoK), which connect neighboring alveoli. A conidium is located on an AEC, which in response releases chemokines with secretion rate $s_{AEC}$ that diffuse on the inner surface of the alveolus with diffusion coefficient *D*. AM follow the chemokine gradient directing their migration towards the AEC with the conidium, which is modeled by a biased persistent random walk (chemotactic migration) with speed $v = 4\;\mu {{{\mathrm{m}}}}/{{{\mathrm{min}}}}$ and persistence time $t_p = 1\;{{{\mathrm{min}}}}$^56,57^. Chemokine secretion and diffusion is modeled by a partial differential equation, while the uptake of chemokines by AM is modeled using a receptor-ligand model based on ordinary differential equations^57,79,80^. Furthermore, PoK as well as the alveolar entrance ring constitute the boundaries of the system and serve as sinks for the diffusing chemokines as well as entry and exit sites for AM. The human and murine alveolus are based on the same model, with model parameters obtaining different values based on literature data that can be found in Supplementary Table 2. For a more detailed description of the model implementation we refer to the Supplementary Material section 1.1 and our previous publications^56–59^.

We give an overview on the computational effort of our vectorized and highly parallelized hABM by the following numbers: On an AMD EPYC 7742 64 core processor, it can take up to one hour to generate a single *IS* data point (5000 simulations), which depends on the values for the diffusion coefficient *D* that is related to the time step $\Delta t$. Using several machines with multiple processors, the total computation time for all simulations was around one and a half months.

### Training a surrogate infection model on hABM simulation data

In addition to the computational analysis of the infection score *IS*, we modeled the infection dynamics also analytically by probabilistic modeling. We found that the individual clearance times *CT* for every parameter configuration follow a Weibull distribution. This distribution follows the characteristics of a reversed Kaplan-Meier curve^81^ that is associated with survival processes or failure times^71^ (see Supplementary Figs. 11 and 12). In the present context, this distribution corresponds to the time-dependent probability that all conidia in an alveolus have been encountered. Thus, the clearance time is a Weibull distributed random variable $CT\sim {{{\mathrm{weib}}}}\left( {\lambda ,k} \right)$and the *IS* is obtained from integrating the probability density function of the Weibull distribution for $CT \,>\, t$:

5 $$IS = P\left( {CT \,>\, t} \right) = {\int}_t^\infty {\lambda k\left( {\lambda x} \right)} ^{k - 1}e^{ - \left( {\lambda x} \right)^k}dx = \left[ { - e^{ - \left( {\lambda x} \right)^k}} \right]_t^\infty = e^{ - \left( {\lambda t} \right)^k}.$$

Since we set the critical time *t* to the constant value $t = 6h$, it is convenient to define $\lambda ^\prime : = \left( {\lambda t} \right)^k$. Using Eq. (5), we determine $\lambda ^\prime = - \ln \left( {IS} \right)$ from the infection score, which is obtained as the numerical result of our simulations by the hABM. Note, that $\lambda ^\prime$ is individually determined for each parameter configuration. It follows, there exists a $\lambda ^\prime$ such that $\lambda ^\prime = \Lambda ^\prime n_{AM}$ for each $n_{AM}$ for the WSM parameter ${{\Lambda }}^\prime$ derived in the Supplementary Material section 1.3.

As can be seen in Supplementary Fig. 6, a superlinear relation of $\lambda ^\prime$ as a function of $n_{AM}$ exists suggesting to approximate $\lambda ^\prime \approx \beta \;{n_{AM}}^\gamma$ for parameters *β* and *γ* that depend on the parameter values $\left( {n_{Con},s_{AEC},D} \right)$. This leads to the compressed exponential function (CEF)

6 $$e^{ - \beta \;n_{AM}^\gamma } \approx e^{ - \lambda ^\prime } = IS$$

and relates the infection score *IS* with the AM number $n_{AM}$. The distributions of the CEF parameters *β* and *γ* are shown in Supplementary Fig. 7 along the ratio *S*_*AEC*_/*D* and can be approximated by a combination of logistic functions $f_{x = \beta ,\;\gamma }:{\Bbb R}^3 \to {\Bbb R}$,

7 $$f_{x = \beta ,\;\gamma }\left( {n_{Con},\;s_{AEC},\;D;\Theta _x} \right) = \left( {\frac{{x_1}}{{1 + \left( {x_2\frac{{s_{AEC}}}{D}} \right)^{x_4}}} - \frac{{x_1}}{{1 + \left( {x_3\frac{{s_{AEC}}}{D}} \right)^{x_4}}}} \right)n_{Con}^{x_6} + x_5n_{Con}^{x_7}$$

for parameters ${{\Theta }} = \left( {{{\Theta }}_\beta ,{{\Theta }}_\gamma } \right),{{\Theta }}_{x = \beta ,\;\gamma } = \left( {x_1, \ldots ,x_7} \right)$. Equation (7) describes a logistic increase and decrease along $s_{AEC}/D$(parameters $x_1,...,x_4$) and is shifted depending on $n_{Con}$ (parameters $\left. {x_5, \ldots ,x_7} \right)$ to fit the distributions of the CEF parameters *β* and *γ* (see Supplementary Fig. 7). Based on our fitting results, we set $\beta \approx f_\beta$ and $\gamma \approx f_\gamma$ in Eq. (6) to derive our *surrogate infection model* ${{{\mathrm{SIM}}}}:{\Bbb R}^4 \to [0,1]$,

8 $${{{\mathrm{SIM}}}}\left( {p;\Theta } \right)^{s = H,M}: = e^{ - f_\beta \left( {n_{Con},\;s_{AEC},D;\Theta _\beta } \right)\;{n_{AM}}^{f_\gamma \left( {n_{Con},\;s_{AEC},D;{{\Theta }}_\gamma } \right)}} \approx IS$$

for the infection score $IS$ depending on the input parameter configurations $p_i^{s = H,M}: = \left( {n_{AM},n_{Con},s_{AEC},D} \right)_i^{s = H,M}$. The identifiability of the SIM is examined in the Supplementary Material section 1.4. The SIM parameters Θ^*s*=*H,M*^ = (Θ_*β*_, Θ_*γ*_)^*s*=*H,M*^ are calibrated by minimizing the mean squared error ($MSE$) between the infection score $IS_{p_i}^{s = H,M}$ as obtained from the simulations with the hABM and the value $IS$ as predicted by the SIM for each parameter configuration $p_i^{s = H,M}$:

9 $$\mathop {{\min }}\limits_\Theta MSE_{{{{\mathrm{SIM}}}}}\left( \Theta \right),\;MSE_{{{{\mathrm{SIM}}}}}\left( \Theta \right) = \frac{1}{N}\mathop {\sum}\limits_i^N {\left( {IS_{p_i}^{s = H,M} - {{{\mathrm{SIM}}}}\left( {p_i;\;\Theta } \right)^{s = H,M}} \right)} ^2,\;N = n^H,\;n^M$$

A schematic overview of the fitting process can be found in the bottom left corner of the Supplementary Fig. 1. After calibration of the SIM parameters Θ^*s*=*H,M*^ for both systems, we are in the position to interpolate the infection score $IS$ for any continuous input parameter configuration *p*.

The mean absolute deviation $MAD_{{{{\mathrm{SIM}}}}}\left( \Theta \right) = \frac{1}{N}\mathop {\sum}\nolimits_i^N {\left| {IS_{p_i}^{s = H,M} - {{{\mathrm{SIM}}}}\left( {p_i;\Theta } \right)} \right|}$ between the $IS_{p_i}^{s = H,M}$ as obtained from all simulation outcomes (infected / not infected) with the hABM and the predicted *IS* is used to evaluate the SIM. An *MAD* < 0.01 suggests a correct SIM prediction of more than 99% of the simulation outcomes and is therefore indicative for a very good agreement between numerical simulations and analytical prediction.

### Reporting summary

Further information on research design is available in the Nature Research Reporting Summary linked to this article.

## Supplementary information

Supplementary Material

Supplementary Video 1

Supplementary Video 2

Supplementary Video 3

Reporting Summary

## Data Availability

The raw simulation data from the hABM as well as Figures and Supplementary Figures and Videos supporting the conclusions of this manuscript can be accessed here: https://asbdata.hki-jena.de/SafferEtAl2022_NPJSBA.

## Code availability

Any code regarding the hybrid agent-based model (hABM) and the derived surrogate infection model (SIM) supporting the conclusions of this manuscript can be accessed here: https://github.com/applied-systems-biology/hABM-AlveolusModel.
