# Supplementary material for: Surrogate infection model predicts optimal alveolar macrophage number for clearance of Aspergillus fumigatus infections
Source: NPJ Syst Biol Appl. 2023 Apr 10;9:12. doi: 10.1038/s41540-023-00272-x (PMC10086013; doi:10.1038/s41540-023-00272-x)
Supplement: Supplementary file 1 — Supplementary Material [file 41540_2023_272_MOESM1_ESM.pdf]

Supplementary Material

Surrogate infection model predicts optimal alveolar macrophage number for clearance of *Aspergillus fumigatus* infections

Christoph Saffer, Sandra Timme, Paul Rudolph and Marc Thilo Figge*

*Correspondence: Corresponding Author thilo.figge@leibniz-hki.de

1. Supplementary Methods

1.1. Hybrid agent-based model (hABM)

With regard to the hABM, we refer to our previous publications where the model was developed for the human[1] alveolus, extended by chemokine diffusion[2], compared with the murine system[3] and studied with special focus on the role of the pores of Kohn (PoK)[4]. The model involves a to-scale three-dimensional representation of a single alveolus, including details such as alveolar epithelial cells (AEC) type I and II, and allows to perform realistic simulations of the early immune response to *Aspergillus fumigatus* infections in the human and murine lung. The model includes two different levels: (i) the cellular level, where cell movements and interactions were realized in continuous space and (ii) the molecular level, where the diffusion of the chemokine concentration was modeled on a lattice by a system of partial differential equations, highly dependent on time, space and the corresponding diffusion parameters s_{AEC}, D, directing immune cell migration. Both levels are realized on a two-dimensional alveolar spherical surface embedded in three-dimensional space. Furthermore, the hABM includes an interface between the two levels allowing for molecule-cell-interactions, which is realized by a set of ordinary differential equations modeling receptor-ligand binding dynamics and. Hence, it enables the included cells to sense molecule gradients caused by secreting AEC, which lets the AM perform a biased random walk towards the conidium to detect the source of infection.

1.2. Calibration of AM input rate λ_{in} in hABM

In the current study, we adjusted the hABM to a varying number of alveolar macrophages (AM). During a simulation AM continuously exit the alveolus randomly through the alveolar entrance ring as well as the pores of Kohn. To guarantee a constant average AM number in the alveolus, we have to add AM and we assume these arrival times t_{wait} to be exponentially distributed for a parameter λ_{in}, which needs to be calibrated for each AM number n_{AM}. It

determines the waiting time t_{wait} until a new AM enters the alveolus, generated by $t_{wait} = 1/\lambda_{in}\ ln(1/u)$ with u drawn from a uniform distribution in $(0,1]$[3,4]. Thus, we calibrated λ_{in} for each AM number $2,4,\dots 50$ in the human alveolus and for $0.1, 0.2, \dots 2.5$ in the murine alveolus. To accomplish the calibration of λ_{in}, we performed 1000 simulations for the configuration with no conidia in the alveolus and an integer number n_{AM} of AM. The AM number is kept constant throughout the simulations by adding an AM whenever an AM did randomly leave the alveolus. We recorded the number of times that AM were added and computed the average number over all simulations to calculate the expected waiting time $E[t_{wait}]$ for a new AM to enter the system. Since we found that t_{wait} is exponentially distributed[1], the reciprocal of the expected arrival time $E[t_{wait}]$ is equal to the parameter $\lambda_{in} = 1/E[t_{wait}]$. Moreover, we used the linear relationship between the parameter λ_{in} and n_{AM} to interpolate the λ_{in} parameter for non-integer numbers n_{AM} in the murine system. After the calibration, we verified the derived λ_{in} parameters by measuring the number of AM throughout simulations for the healthy state by comparison to the expected number of AM.

1.3. Weibull Survival Model (WSM)

For a system with n_{AM} AM searching for one conidium we assume $X_1, \dots, X_{n_{AM}}$ equal Weibull distributed random variables X_i describing the clearance times (CT), i.*e.* the time until the i–th AM finds the conidium. This is concluded from the Weibull distributed CT shown in Supplementary Figures 11 and 12. Under the assumption of *independence* of these events, their joint distribution is given as:

$$IS = P(CT > t) = P(X_1, \dots, X_{n_{AM}} > t) = \prod_{i=1,\dots,n_{AM}} P(X_i > t) = e^{-(\Lambda t)^K n_{AM}} \quad (1)$$

There are the shape parameter Λ and scale parameter K, which determines if the event occurs more often $(K > 1)$ or less often $(K < 1)$ over time[5]. The condition $X_1^j, \dots, X_{n_{AM}}^j > t$ is equal to $Y_j > t$ for $Y_j = \min(X_1^j, \dots, X_{n_{AM}}^j)$ describing the time until the $j-th$ conidia was detected by an AM for the first time (minimal CT). Under the assumptions of equal Weibull distributed random variables X_i^j and independence of these events in the case of finding n_{Con} conidia, we formulate the infection score for fixed input parameters s_{AEC}, D and n_{AM}, n_{Con}:

$$IS = P(CT > t) = 1 - P(CT < t) = 1 - P(Y_1, \dots Y_{n_{Con}} < t) = 1 - \prod_{j=1,\dots,n_{Con}} P(Y_j < t) \quad (2)$$

$$= 1 - \prod_{j=1,\dots,n_{Con}} P(X_1^j, \dots, X_{n_{AM}}^j < t) = 1 - \prod_{j=1,\dots,n_{Con}} (1 - e^{-(\Lambda t)^K n_{AM}}) \quad (3)$$

$$= 1 - \left(1 - e^{-(\Lambda t)^K n_{AM}}\right)^{n_{Con}} = 1 - \left(1 - e^{-\Lambda' n_{AM}}\right)^{n_{Con}} \quad (4)$$

Since $t = 6h$, we set $(\Lambda t)^K \coloneqq \Lambda'$ and adapted the model as a function of varying numbers of searching agents n_{AM} and targets n_{Con} but for a fixed time t. This model is also applied for modeling searching or failure processes in parallel systems[5] as a function of time t.

1.4. Identifiability of the SIM

A model M with parameters Θ is identifiable when there is a one-to-one mapping from $\Theta \mapsto M$. For sets of parameters Θ, Θ', we show that from $M(;\Theta) = M(;\Theta')$ follows $\Theta = \Theta'$. To show the identifiability of the SIM, we show the identifiability of its three submodels:

I) $SIM(n_{AM}, n_{Con}, s_{AEC}, D; \Theta_\beta, \Theta_\gamma) = e^{-f(n_{Con}, s_{AEC}, D;\, \Theta_\beta) n_{AM}{}^{f(n_{Con}, s_{AEC}, D;\, \Theta_\gamma)}}$

II) $f(n_{Con}, s_{AEC}, D;\ \Theta_x) = L(Z; x_1 \dots, x_4)\, n_{Con}{}^{x_6} + x_5\, n_{Con}{}^{x_7}$ for $Z \coloneqq s_{AEC}/D$

III) $L(Z\,; x_1 \dots, x_4) = \frac{x_1}{1+(x_2\, Z)^{x_4}} - \frac{x_1}{1+(x_3\, Z)^{x_4}}$, for $x_2 \neq x_3$

I) Let $\Theta_\beta, \Theta_\gamma$ and $\Theta'_\beta, \Theta'_\gamma$ be parameters for f. For all $n_{AM}, n_{Con}, s_{AEC}, D$, $f(\dots;\ \Theta'_\beta) \neq 0$ applies:

$$e^{-f(\dots;\,\Theta_\beta) n_{AM}{}^{f(\dots;\,\Theta_\gamma)}} = e^{-f(\dots;\,\Theta'_\beta) n_{AM}{}^{f(\dots;\,\Theta'_\gamma)}} \tag{5}$$

$$\Leftrightarrow f(\dots;\ \Theta_\beta) n_{AM}{}^{f(\dots;\,\Theta_\gamma)} = f(\dots;\ \Theta'_\beta) n_{AM}{}^{f(\dots;\,\Theta'_\gamma)} \tag{6}$$

$$\Leftrightarrow \frac{f(\dots;\,\Theta_\beta)}{f(\dots;\,\Theta'_\beta)} = n_{AM}{}^{f(\dots;\,\Theta'_\gamma) - f(\dots;\,\Theta_\gamma)} \tag{7}$$

Equation (7) is true if $f(\dots;\ \Theta_\beta) = f(\dots;\ \Theta'_\beta)$ and $f(\dots;\ \Theta_\gamma) = f(\dots;\ \Theta'_\gamma)$ for all $n_{AM}, n_{Con}, s_{AEC}, D$. Now, it is left to show, that $f(\dots; \Theta_x) = f(\dots; \Theta'_x) \Leftrightarrow \Theta_x = \Theta'_x$.

II) Let Θ_x and Θ'_x be parameters for f. For all n_{Con}, Z and $x_5 \neq 0, L \neq 0$ applies:

$$L(Z; x_1 \dots, x_4)\, n_{Con}{}^{x_6} + x_5\, n_{Con}{}^{x_7} = L(Z; x'_1 \dots, x'_4)\, n_{Con}{}^{x'_6} + x'_5\, n_{Con}{}^{x'_7} \tag{8}$$

$$\Leftrightarrow L(Z; x_1 \dots, x_4)\, n_{Con}{}^{x_6} - L(Z; x'_1 \dots, x'_4)\, n_{Con}{}^{x'_6} = x_5\, n_{Con}{}^{x_7} - x'_5\, n_{Con}{}^{x'_7} \tag{9}$$

This can only be true for all n_{Con}, Z if both sides of equation (9) are zero because the right side does not depend on Z. Then it directly follows from I) that $x_5 = x'_5, x_6 = x'_6, x_7 = x_7{}'$ and $L(Z; x_1 \dots, x_4) = L(Z; x'_1 \dots, x'_4)$. In the next step it is left to show that for all : $L(Z; x_1 \dots, x_4) = L(Z; x_1{}' \dots, x_4{}') \Leftrightarrow x_1 = x'_1, x_2 = x'_2, x_3 = x'_3, x_4 = x'_4$.

III) Let x_1, x_2, x_3, x_4 and x'_1, x'_2, x'_3, x'_4 be parameters for L. For all Z and $x_2 \neq x_3$ applies:

$$\frac{x_1}{1+(x_2\, Z)^{x_4}} - \frac{x_1}{1+(x_3\, Z)^{x_4}} = \frac{x'_1}{1+(x'_2\, Z)^{x'_4}} - \frac{x'_1}{1+(x'_3\, Z)^{x'_4}} \tag{10}$$

Through numerical simulations, we find parameter mappings $x_1 = -x'_1, x_2 = x'_3, x_3 = x'_2, x_4 = x'_4$ and $x_1 = x'_1, x_2 = -x'_3, x_3 = -x'_2, x_4 = -x'_4$ that violate the identifiability. These mappings

cause a switch in the sign and the order of the logistic functions, but produce identical results. To prevent this, we verify numerically L to be identifiable for $x_1, x_4 > 0$.

For $x_1, x_4 > 0$ identifiability of III) $\Longrightarrow$ identifiability of II) $\Longrightarrow$ identifiability of I) $\Longrightarrow$ SIM is identifiable for $x_1, x_4 > 0$.

1.5. Model validation

The performance of the surrogate infection model (SIM) was compared to state-of-the-art machine learning models: a multilayer perceptron network (MLP)[6] from the class of the feedforward artificial networks and a random decision forest (RDF)[7]. Both models take the same input variables $n_{AM}, n_{Con}, s_{AEC}/D$ as the SIM and predict the infection score IS. The MLP was optimized and tested for different numbers of layers and parameters until we found the best configuration in terms of the MAD for two hidden layers with 13 and 3 neurons, respectively, and 98 parameters in total, where an activation function of sigmoidal shape was used. We repeated the procedure for the RDF and found the best configuration in terms of the MAD with about 100 subtrees. All three models were compared using five times repeated 6-fold cross validation[8], where the data set was split into six subsets and trained on five on them, while test scores were derived on the 6th data set. We repeated the training and prediction six times until each subset was test set. The entire procedure was repeated five times to prevent validation results biased by one random split. Ultimately, we received 30 predictions on train and test sets for each of the three models as shown in Figure 5 and bottom right corner of Supplementary Figure 1.

1.6. Weighted minimum

Following Blickensdorf *et. al.*[3], we computed the weighted minimum of the infection score IS for each tupel of diffusion coefficient and secretion rate $(D_j, s_{AEC_j})$with values $D_j \in \{20, 60, \dots, 6000\}$ and $s_{AEC_i} \in \{1\,500, 5\,000, \dots, 500\,000\}$. Taking only ratios s_{AEC_i}/D_j into account where the respective $IS_{s_{AEC_i}, D_j}$ is within the 95% confidence interval of the optimal IS, the weighted minimum of the ratios s_{AEC}/D was calculated as $s_{AEC}/D_{opt} = \frac{1}{\sum_{i,j} w_{i,j}} \sum_{i,j} \frac{1}{w_{i,j}} s_{AEC_i}/D_j$ with weights $w_{i,j} = 1 - IS_{s_{AEC_i}, D_j}$. This was applied for each of the 25 screened AM numbers n_{AM} and each fungal burden for the human and murine system (see Figure 6 and Supplementary Figure 10).

1.7. Programming languages

The hABM of the human and murine alveolus was fully implemented in modern C++[1,2]. Data analysis and fitting of analytical models were done in *Python 3.8* using the packages *Numpy* and *Pandas* for data analysis and the *Scipy* packages for optimization. The MLP and RDF were implemented using *PyTorch* and *Scikit-Learn,* respectively.

1.7 References

1. Pollmächer, J. & Figge, M. T. Agent-based model of human alveoli predicts chemotactic signaling by epithelial cells during early Aspergillus fumigatus infection. *PLoS One* **9**, (2014).

2. Pollmächer, J. & Figge, M. T. Deciphering chemokine properties by a hybrid agent-based model of Aspergillus fumigatus infection in human alveoli. *Front. Microbiol.* **6**, 1–14 (2015).

3. Blickensdorf, M., Timme, S. & Figge, M. T. Comparative Assessment of Aspergillosis by Virtual Infection Modeling in Murine and Human Lung. *Front. Immunol.* **10**, (2019).

4. Blickensdorf, M., Timme, S. & Figge, M. T. Hybrid Agent-Based Modeling of Aspergillus fumigatus Infection to Quantitatively Investigate the Role of Pores of Kohn in Human Alveoli. *Front. Microbiol.* **11**, 1–13 (2020).

5. McCool, J. I. *Using the Weibull Distribution: Reliability, Modeling, and Inference* 73-82 (Wiley, 2012).

6. Schmidhuber, J. Deep Learning in neural networks: An overview. *Neural Networks* **61**, 85–117 (2015).

7. Liaw, A. & Wiener, M. Classification and Regression by randomForest. *R News* **2**, 18–22 (2002).

8. Vanwinckelen, G. & Blockeel, H. On estimating model accuracy with repeated cross-validation. In: *21st Belgian-Dutch Conf. on Machine Learning* 39–44 (Ghent University, 2012)

2. Supplementary Videos

Supplementary Videos can be accessed here:
https://asbdata.hki-jena.de/SafferEtAl2022_NPJSBA

2.1. Supplementary Video 1: Infection scenarios in human alveolus

The video shows exemplary infection scenarios for four different parameter configurations in the human alveolus.

2.2. Supplementary Video 2: Infection scenarios in murine alveolus

The video shows exemplary infection scenarios for four different parameter configurations in the murine alveolus.

2.3. Supplementary Video 3: Comparison of SIM output in humans and mice

Comparison of infection scores predicted by the surrogate infection model (SIM) for an increasing ratio of s_{AEC}/D for the human (green) and murine (orange) system for low (dark colors) and high (bright colors) fungal burden. Grey dashed vertical lines denote the 5% and 10% ASC for both organisms. Colored dashed vertical lines represent the corresponding AM numbers as obtained by literature data.

3. Supplementary Figures

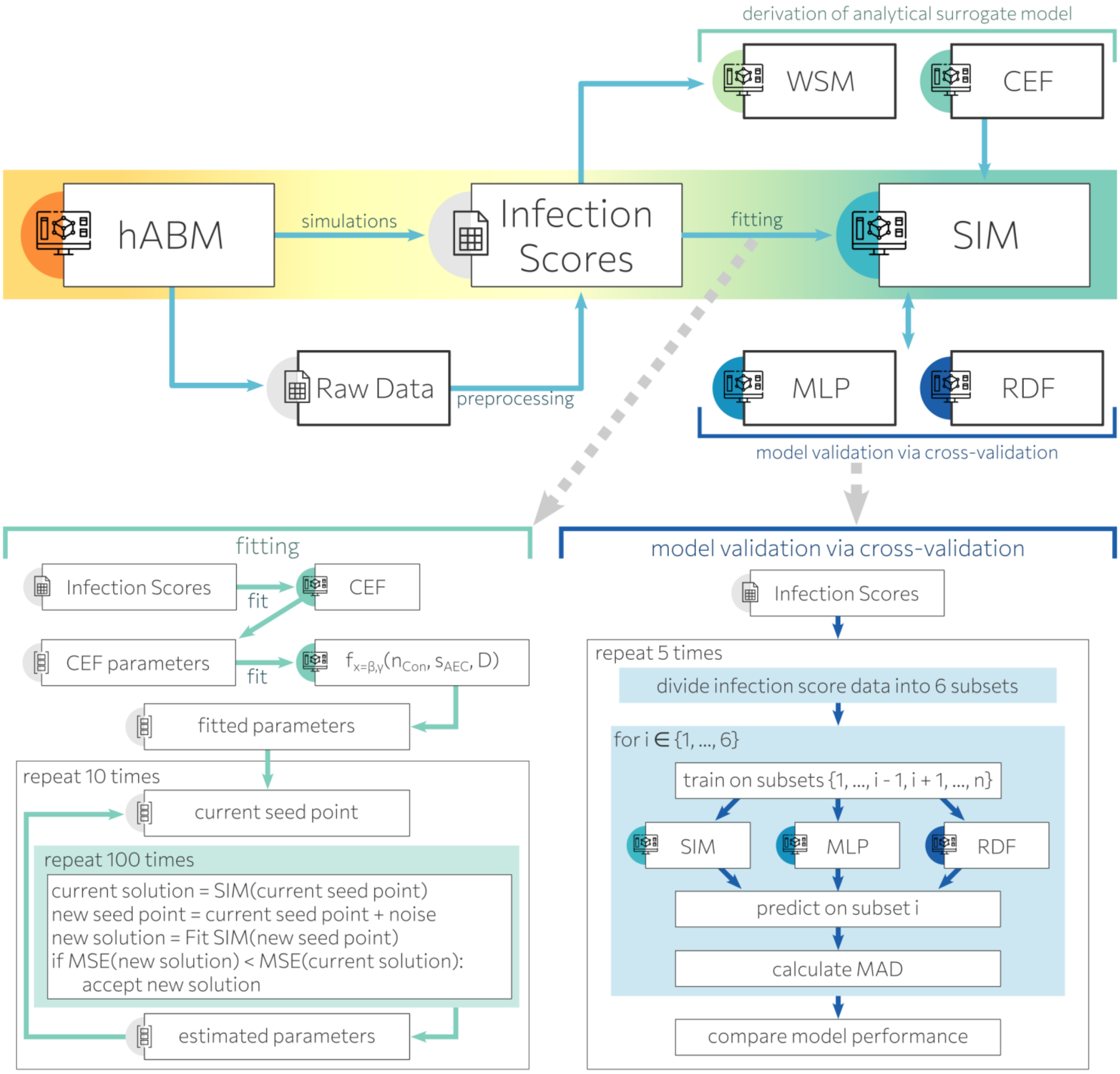

Supplementary Figure 1: A scheme of the entire workflow is presented: The hABM is applied to run millions of simulations to predict infection scores (IS) for different parameter configurations. From these IS, we derived a Weibull Survival Model (WSM) for low fungal burden and extend this to the Compressed Exponential Function (CEF) for high fungal burden. Based on this, we developed a surrogate infection model (SIM); *i.e.* an analytical model that predicts infection scores of the hABM for various parameter configurations in a fraction of time. In the bottom left corner, the fitting algorithm of the SIM to the IS is depicted. In the bottom right corner, the 5 times 6-fold cross validation is shown to compare the performance of the SIM with state-of-the-art machine learning models, *i.e.* the multilayer perceptron (MLP) and a random decision forest (RDF).

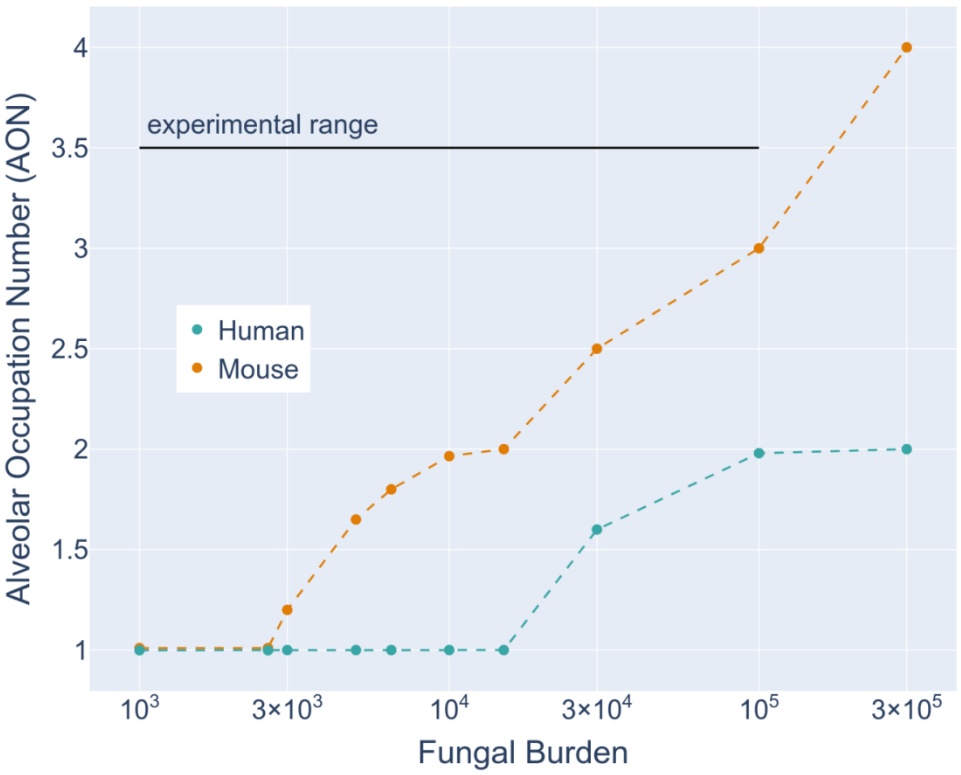

Supplementary Figure 2: Expected alveolar occupation number as a function of the fungal burden in the human (green) and murine (orange) alveolus.

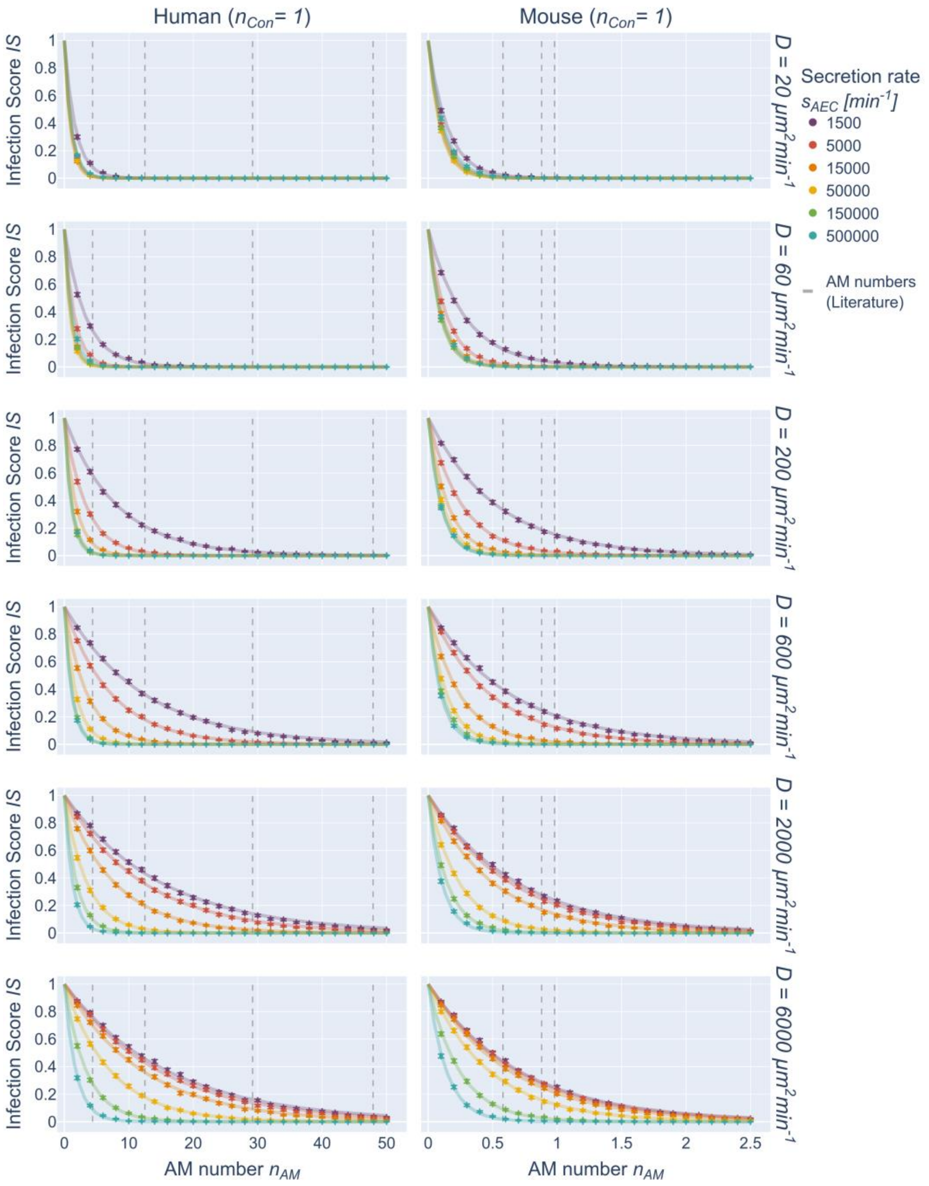

Supplementary Figure 3: Simulated data from the hABM are shown as points for low fungal burden in the human (left row) and murine (right row) alveolus for $D = (20,\ 60,\ 200,\ 600,\ 2\,000,\ 6\,000)\ \mu m^2/min$ (panels from top to bottom) and secretion rates $s_{AEC} = (1\,500,\ 5\,000,\ 15\,000,\ 50\,000,\ 150\,000,\ 500\,000)\ min^{-1}$ (different colors). Error bars represent the 95% confidence interval as obtained from the standard error of independent Bernoulli trials. Solid lines represent the fit to the data points as obtained by the Weibull survival model (WSM). Dashed vertical lines denote AM numbers from literature.

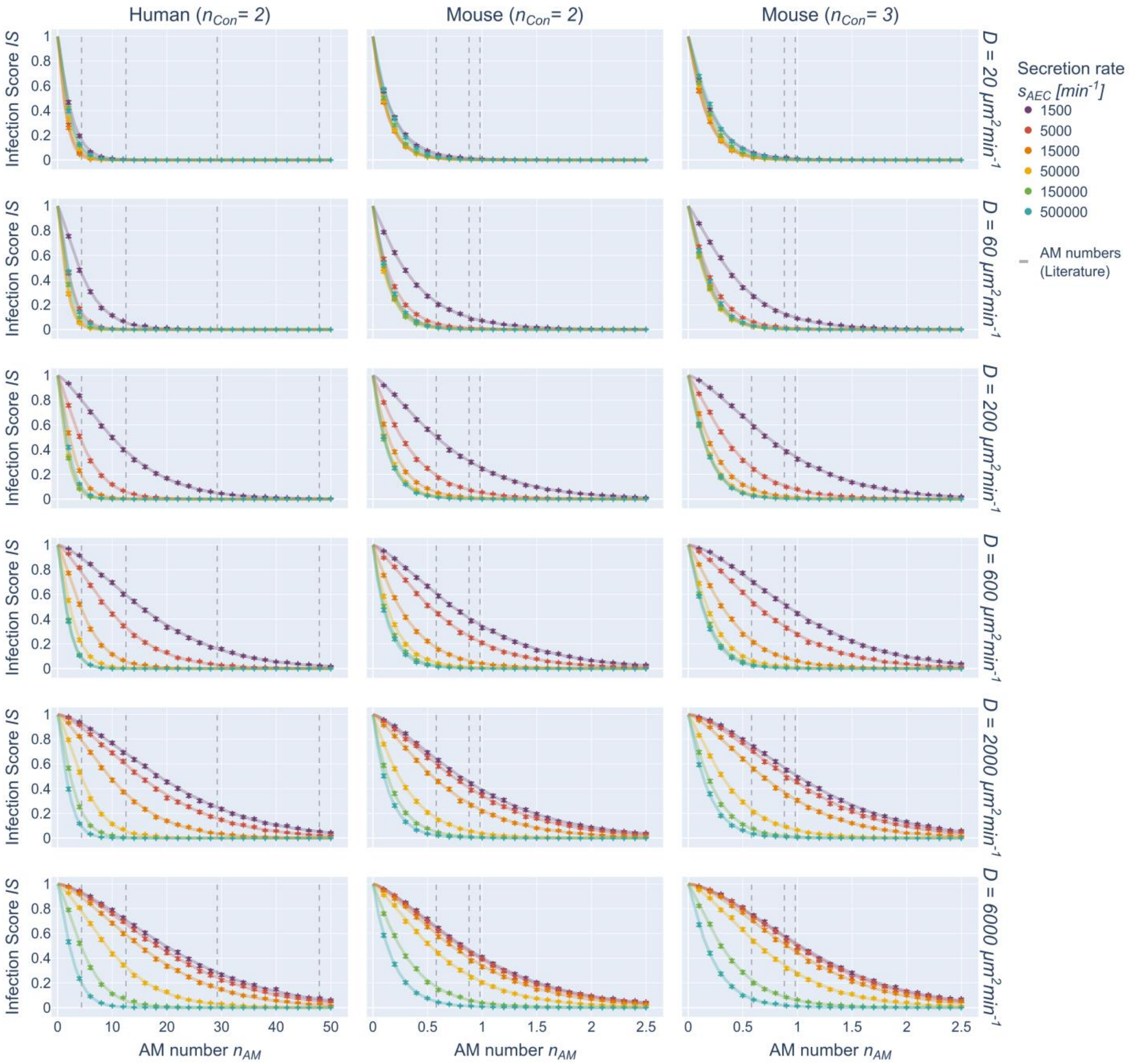

Supplementary Figure 4: Simulated data from the hABM are shown as points for high fungal burden in the human and murine alveolus for $D = (20,\ 60,\ 200,\ 600,\ 2\,000,\ 6\,000)\ \mu m^2/min$ (panels from top to bottom) and secretion rates $s_{AEC} = (1\,500,\ 5\,000,\ 15\,000,\ 50\,000,\ 150\,000,\ 500\,000)\ min^{-1}$ (different colors). Error bars represent the 95% confidence interval as obtained from the standard error of independent Bernoulli trials. Solid lines represent the fit to the data points as obtained by the compressed exponential function (CEF). Dashed vertical lines denote AM numbers from literature.

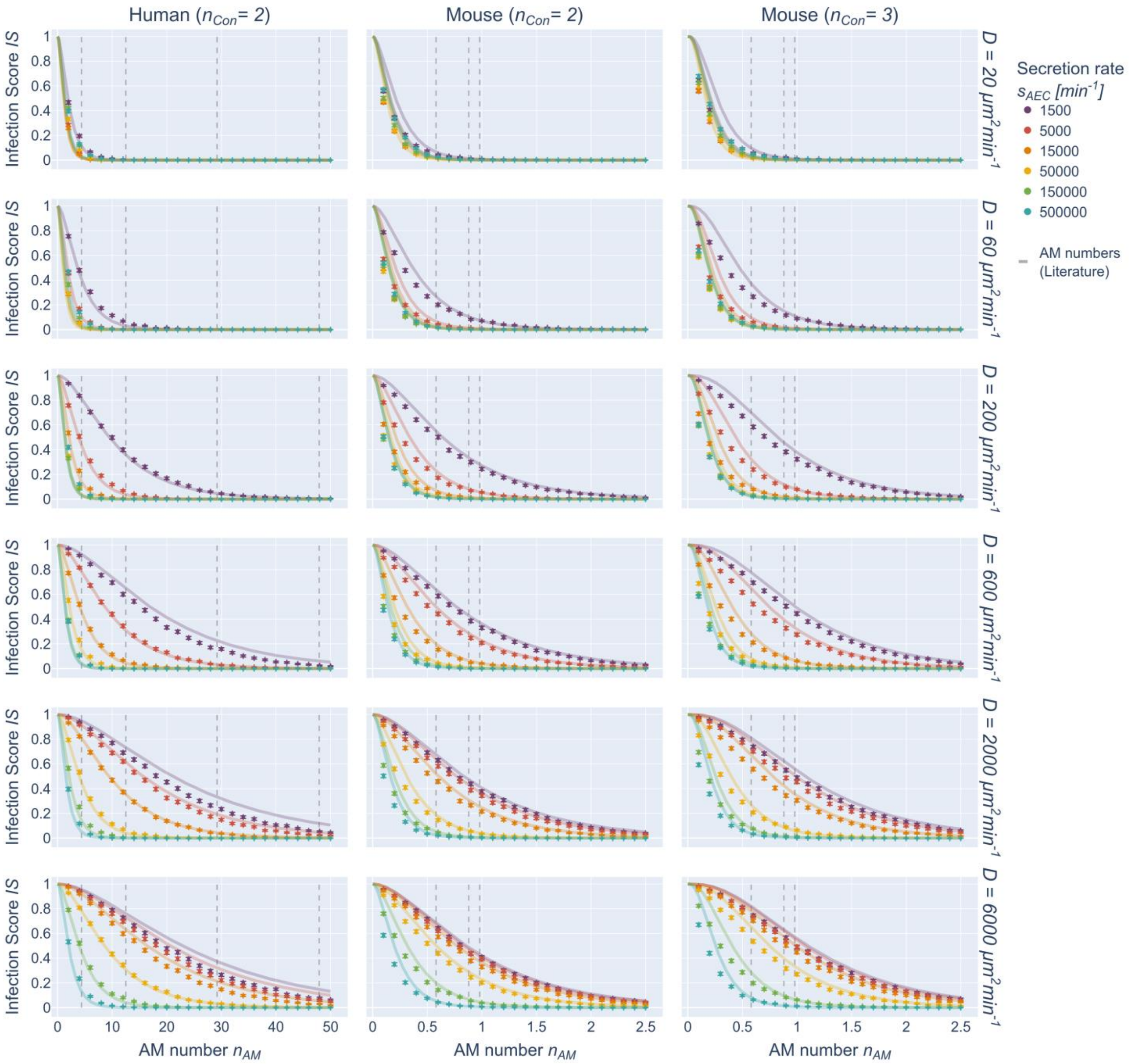

Supplementary Figure 5: Simulated data from the hABM are shown as points for high fungal burden $(n_{Con} > 1)$ in the human and murine alveolus for $D = (20,\ 60,\ 200,\ 600,\ 2\,000,\ 6\,000)\ \mu m^2/min$ (panels from top to bottom) and secretion rates $s_{AEC} = (1\,500,\ 5\,000,\ 15\,000,\ 50\,000,\ 150\,000,\ 500\,000)\ min^{-1}$ (different colors). Error bars represent the 95% confidence interval as obtained from the standard error of independent Bernoulli trials. Solid lines represent the fit to the data points as obtained by the Weibull survival model (WSM). Dashed vertical lines denote AM numbers from literature.

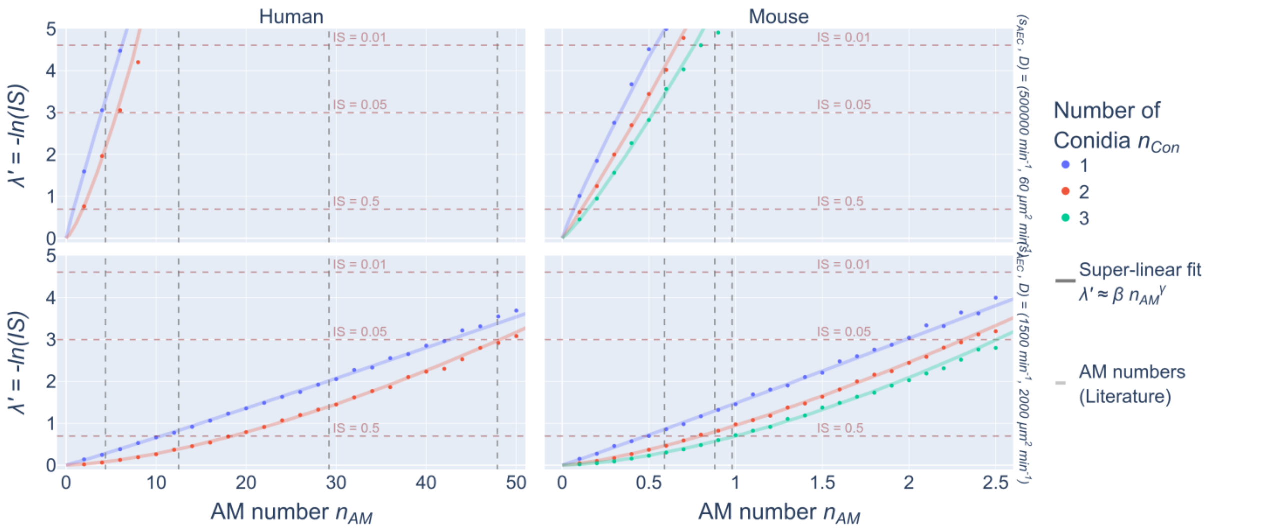

Supplementary Figure 6: Estimated parameters $\lambda' = -\ln(IS)$ (points) over the AM numbers based on simulated data by the hABM approximated by a superlinear function $\lambda' \approx \beta {n_{AM}}^{\gamma}$ (solid lines). Dashed vertical lines denote AM numbers from literature.

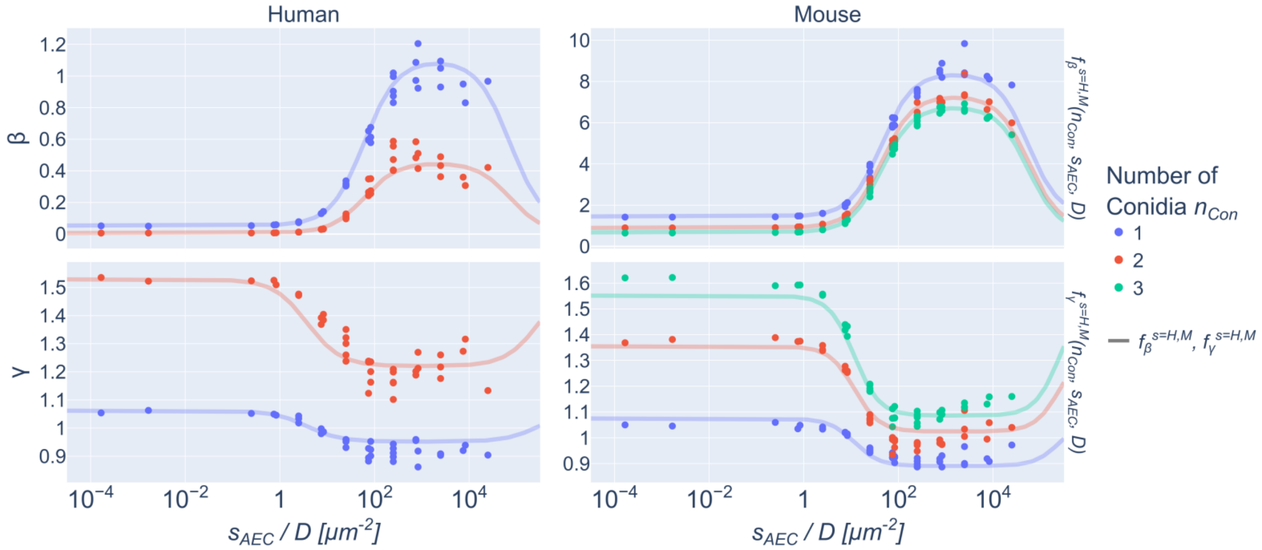

Supplementary Figure 7: Estimated parameter values β and γ (points) over chemokine ratio s_{AEC}/D for superlinear function $\lambda' \approx \beta {n_{AM}}^{\gamma}$ approximated by a combination of logistic functions f_β, f_γ (solid lines). Dashed vertical lines denote AM numbers from literature.

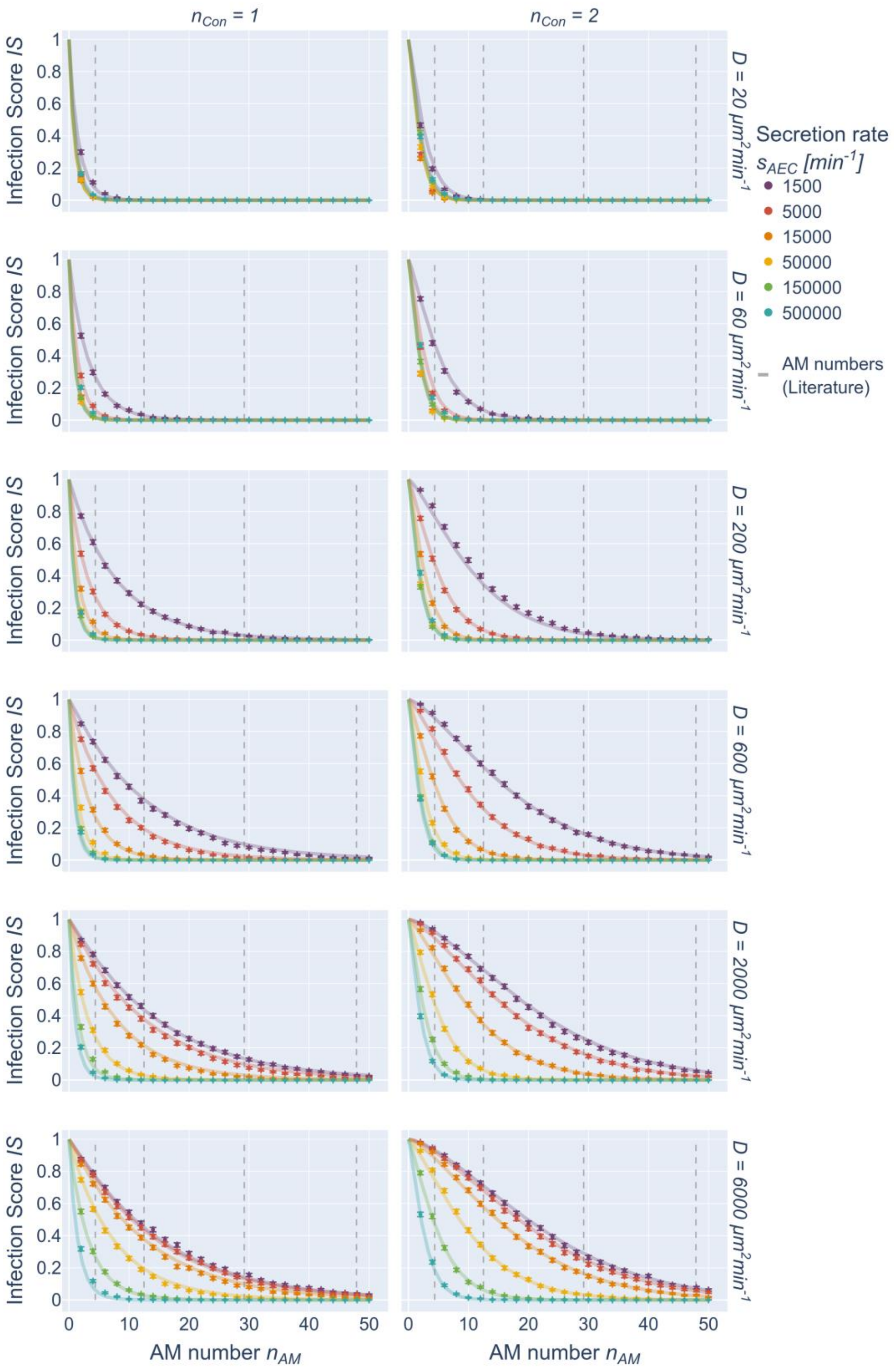

Supplementary Figure 8: Simulated data from the hABM are shown as points for low (left row) and high (right row) fungal burden in the human alveolus for $D = (20,\ 60,\ 200,\ 600,\ 2\,000,\ 6\,000)\ \mu m^2/min$ (panels from top to bottom) and secretion rates $s_{AEC} = (1\,500,\ 5\,000,\ 15\,000,\ 50\,000,\ 150\,000,\ 500\,000)\ min^{-1}$ (different colors). Error bars represent the 95% confidence interval as obtained from the standard error of independent Bernoulli trials. Solid lines represent the fit to the data points as obtained by the surrogate infection model (SIM). Dashed vertical lines denote AM numbers from literature.

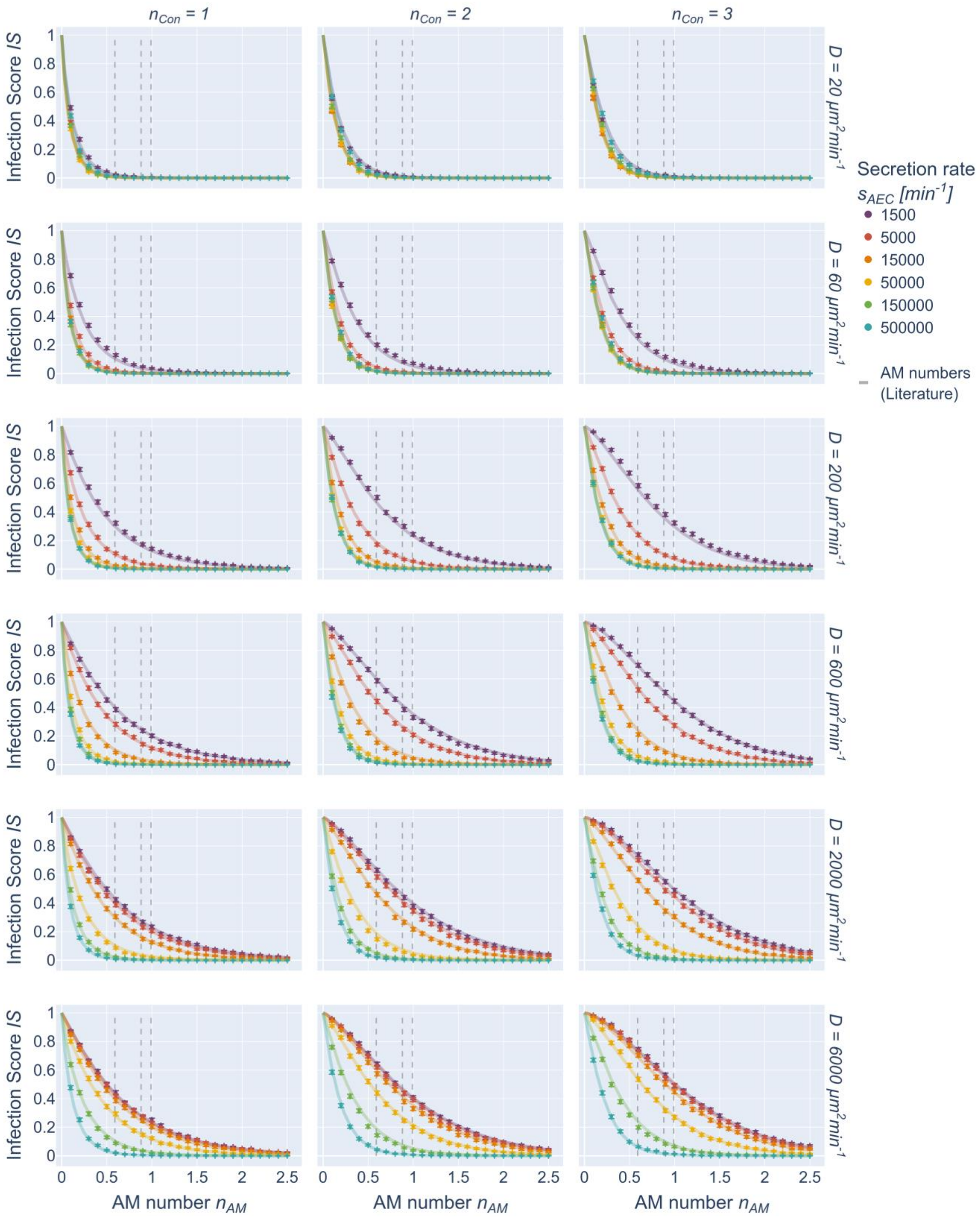

Supplementary Figure 9: Simulated data from the hABM are shown as points for low (left row) and high (right row) fungal burden in the murine alveolus for $D = (20,\ 60,\ 200,\ 600,\ 2\,000,\ 6\,000)\ \mu m^2/min$ (panels from top to bottom) and secretion rates $s_{AEC} = (1\,500,\ 5\,000,\ 15\,000,\ 50\,000,\ 150\,000,\ 500\,000)\ min^{-1}$ (different colors). Error bars represent the 95% confidence interval as obtained from the standard error of independent Bernoulli trials. Solid lines represent the fit to the data points as obtained by the surrogate infection model (SIM). Dashed vertical lines denote AM numbers from literature.

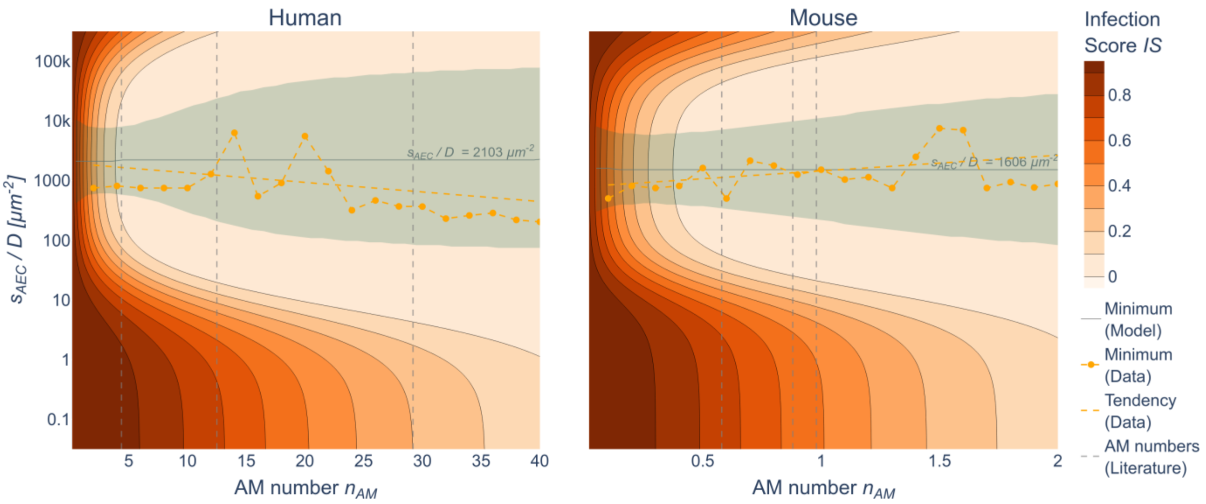

Supplementary Figure 10: Predicted infection scores (orange gradient) by the surrogate infection model (SIM) for pairs of AM numbers n_{AM} and chemokine ratios s_{AEC}/D for high fungal burden in the human (left) and murine (right) alveolus. Dashed vertical lines denote AM numbers from literature. The grey area represents the 95% confidence interval around optimal value s_{AEC}/D for the lowest infection score derived from the SIM. Yellow points represent optimal values derived from the simulation data (yellow dashed lines as a guide for the eye).

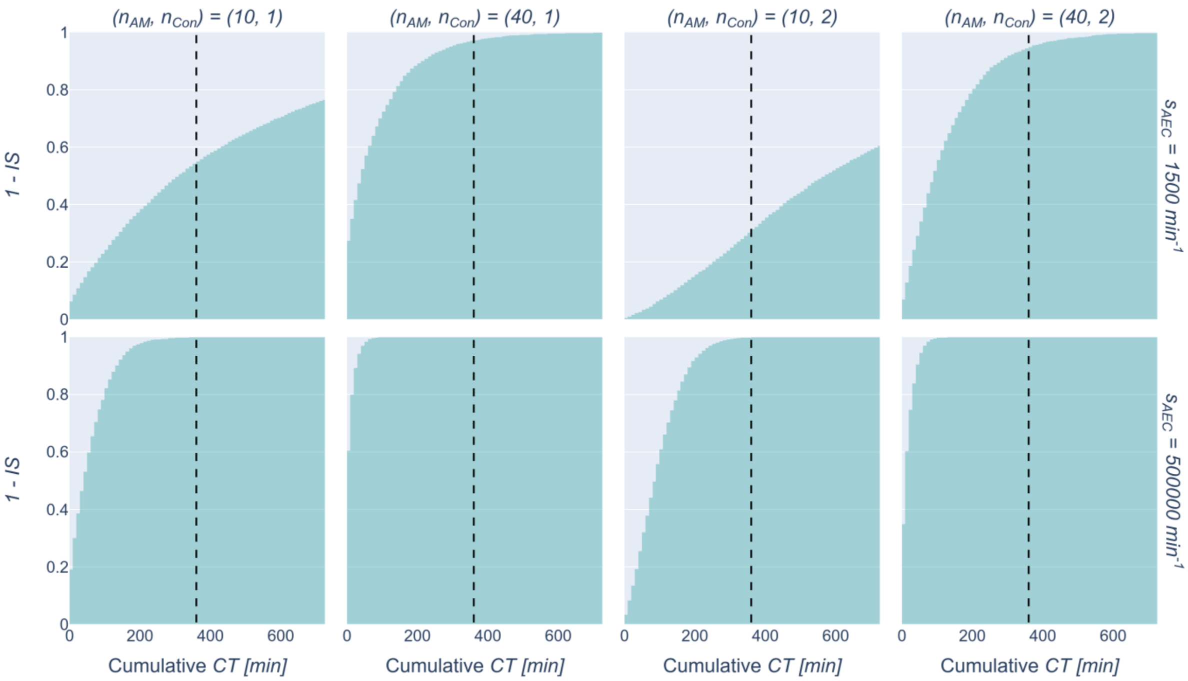

Supplementary Figure 11: Cumulative clearing times (CT) in the human system for $D = 600\ \mu m^2/min$. Black vertical dashed lines denotes the 6h time point, where the onset of hyphal growth can be expected.

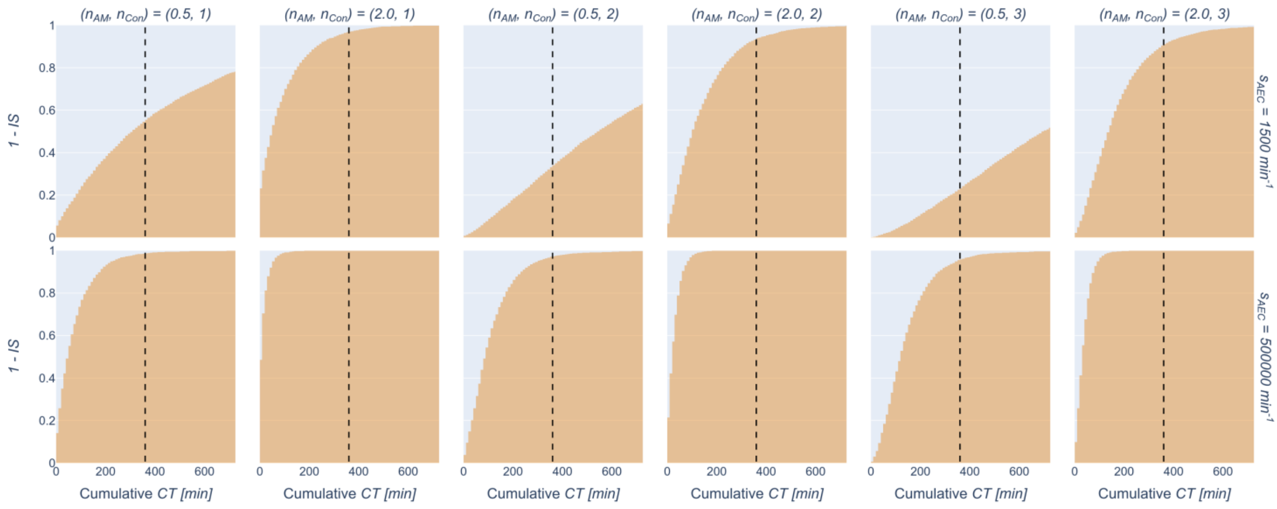

Supplementary Figure 12: Cumulative clearing times (CT) in the murine system for $D = 600\ \mu m^2/min$. Black vertical dashed lines denotes the 6h time point, where the onset of hyphal growth can be expected.

4. Supplementary Tables

Infection score	Human				Mouse			
	qRW		Optimal ratio		qRW		Optimal ratio	
	Low FB	High FB	Low FB	High FB	Low FB	High FB	Low FB	High FB
10^{-1}	31	45	2.3	3.9	1.6	2.2	0.24	0.38
10^{-2}	65	71	4.7	6.9	3.0	3.5	0.52	0.71
10^{-3}	97	92	7.1	9.5	4.3	4.5	0.82	1.03
10^{-4}			9.6	12.0			1.13	1.35
10^{-5}			12.1	14.5			1.45	1.65
10^{-6}			14.6	16.8			1.78	1.95

Supplementary Table 1: From Figure 7 extracted AM numbers required to reach certain threshold values of infection clearance that are measured by the infection scores in human / mouse for optimal chemokine ratios / qRW and a low / high fungal burden. Colored cells denote values that are extrapolated by the SIM (red), *i.e.*, $IS < 1/5000 = 0.0002$, or that are outside the ranges of the AM numbers (blue), *i.e.* $n_{AM}^{H} > 50$, $n_{AM}^{M} > 2.5$.

System	Human	Mouse
Number of alveoli	$4.8 \cdot 10^8$	$3.3 \cdot 10^6$
Radius of alveolus	$116.5\ \mu m$	$26.2\ \mu m$
Area of alveolus (3/4 - sphere)	$1.28 \cdot 10^5\ \mu m^2$	$6.46 \cdot 10^3\ \mu m^2$
Radius of AM	$10.6\ \mu m$	$9.5\ \mu m$
Area of AM	$3.53 \cdot 10^2\ \mu m^2$	$2.84 \cdot 10^2\ \mu m^2$
Covered area per AM	$0.276\ \%$	$4.390\ \%$
Speed of AM	$4\ \mu m/min$	$4\ \mu m/min$
Number of AEC type I (II)	$48\ (84)$	$4\ (4)$
Radius AEC type I (edge length AEC type II)	$27\ \mu m\ (9.34\ \mu m)$	$22\ \mu m\ (8.12\ \mu m)$
Number of PoK	24	7

Supplementary Table 2: Average properties derived for the human and murine alveolus.
